# Epidemiology, genetics, and subtyping of preserved ratio impaired spirometry (PRISm) in COPDGene

**DOI:** 10.1186/s12931-014-0089-y

**Published:** 2014-08-06

**Authors:** Emily S Wan, Peter J Castaldi, Michael H Cho, John E Hokanson, Elizabeth A Regan, Barry J Make, Terri H Beaty, MeiLan K Han, Jeffrey L Curtis, Douglas Curran-Everett, David A Lynch, Dawn L DeMeo, James D Crapo, Edwin K Silverman, The COPDGene Investigators

**Affiliations:** Channing Division of Network Medicine, Brigham and Women’s Hospital, 181 Longwood Avenue, 4th floor, Boston, MA 2115 USA; Division of Pulmonary and Critical Care Medicine, Brigham and Women’s Hospital, Boston, MA USA; Department of Epidemiology, Colorado School of Public Health, University of Colorado, Denver, CO USA; National Jewish Health, Denver, CO USA; Department of Epidemiology, Bloomberg School of Public Health, Johns Hopkins University, Baltimore, MD USA; Division of Pulmonary and Critical Care Medicine, University of Michigan Health System, Ann Arbor, MI USA; Pulmonary and Critical Care Section, Ann Arbor Veterans Affairs Healthcare System, Ann Arbor, MI USA; Division of Biostatistics and Bioinformatics, National Jewish Health, Denver, CO USA; Department of Biostatistics and Informatics, Colorado School of Public Health, University of Colorado Denver, Denver, CO USA

**Keywords:** Spirometry, Restriction, Lung diseases, Smoking

## Abstract

**Background:**

Preserved Ratio Impaired Spirometry (PRISm), defined as a reduced FEV_1_ in the setting of a preserved FEV_1_/FVC ratio, is highly prevalent and is associated with increased respiratory symptoms, systemic inflammation, and mortality. Studies investigating quantitative chest tomographic features, genetic associations, and subtypes in PRISm subjects have not been reported.

**Methods:**

Data from current and former smokers enrolled in COPDGene (n = 10,192), an observational, cross-sectional study which recruited subjects aged 45–80 with ≥10 pack years of smoking, were analyzed. To identify epidemiological and radiographic predictors of PRISm, we performed univariate and multivariate analyses comparing PRISm subjects both to control subjects with normal spirometry and to subjects with COPD. To investigate common genetic predictors of PRISm, we performed a genome-wide association study (GWAS). To explore potential subgroups within PRISm, we performed unsupervised k-means clustering.

**Results:**

The prevalence of PRISm in COPDGene is 12.3%. Increased dyspnea, reduced 6-minute walk distance, increased percent emphysema and decreased total lung capacity, as well as increased segmental bronchial wall area percentage were significant predictors (p-value <0.05) of PRISm status when compared to control subjects in multivariate models. Although no common genetic variants were identified on GWAS testing, a significant association with Klinefelter’s syndrome (47XXY) was observed (p-value < 0.001). Subgroups identified through k-means clustering include a putative “COPD-subtype”, “Restrictive-subtype”, and a highly symptomatic “Metabolic-subtype”.

**Conclusions:**

PRISm subjects are clinically and genetically heterogeneous. Future investigations into the pathophysiological mechanisms behind and potential treatment options for subgroups within PRISm are warranted.

**Trial registration:**

Clinicaltrials.gov Identifier: NCT000608764.

**Electronic supplementary material:**

The online version of this article (doi:10.1186/s12931-014-0089-y) contains supplementary material, which is available to authorized users.

## Background

Since its inception in the mid-19th century [1], spirometry has become an accepted tool in the diagnosis and staging of obstructive lung diseases (defined as the disproportionate reduction in the forced expiratory volume in the first second (FEV_1_) relative to the forced vital capacity (FVC)). However, subjects with substantial, *proportionate* impairments in FEV_1_ and FVC resulting in a preserved FEV_1_/FVC ratio have remained a relatively understudied group. Approximately 1 out of every 8 subjects in the general population has *P*reserved *R*atio *I*mpaired *S*piro*m*etry (PRISm); this pattern has alternatively been referred to as “unclassified”, “non-specific”, or “restrictive” spirometry, with the latter term being the most widely accepted. It should be noted, however, that the predictive value of “restrictive spirometry” for true restriction, as defined by a reduced total lung capacity, is poor [2–4]. In addition, although restriction is often associated with interstitial lung diseases, many PRISm subjects do *not* have evidence for interstitial lung diseases on radiographic evaluation [5]. Less commonly used terms, such as ”non-specific” [6] or “unclassified” spirometry [7,8], do not make inferences regarding the etiology of the spirometric abnormalities, but are generally uninformative.

The cross-sectional prevalence of PRISm has been estimated to be between 6.6%-17.6% [9–16] worldwide. While local and regional variability in the prevalence of PRISm exists [13,15], these estimates remain stable regardless of whether the Global Initiative for Obstructive Lung Disease [17] (GOLD) or lower limits of normal (LLN) diagnostic criteria are utilized [8,14,16]. Subjects with PRISm have increased morbidity [8–10,12,15,16,18] and mortality [6,9,16,18]. They report increased respiratory symptoms [9,16,19], decreased exercise capacity [12,19], more difficulty with the activities of daily living [12,15], and have evidence of increased systemic inflammation [20]. Additional trends which have emerged include associations between PRISm and increased body mass index (BMI) [6,9,10,15], diabetes mellitus [8,12,13,18,21], cardiovascular disease [9,13,18,22], and cigarette smoke exposure [6,10,13,15,18]. While these summary statistics among all PRISm subjects are useful, they fail to capture the significant heterogeneity present within this group; for example, while the mean BMI of this cohort is typically higher than that of the general population, the range of BMI observed can include frankly cachectic subjects [8,10,12,13,15,18]. In our previous analysis of PRISm subjects among the first 2,500 subjects from COPDGene [8], we hypothesized that this heterogeneity reflected the multitude of potential underlying causes for this spirometric pattern. Using data from current and former smokers enrolled in the full COPDGene cohort, we now seek to examine the following hypotheses:

- *Aim 1*: We hypothesize that a distinct set of predictors are associated with PRISm status and examine the epidemiological, functional, and radiographic predictors of PRISm status relative to control and COPD subjects.
- *Aim 2*: We hypothesize that genetic variants may contribute to the development of PRISm among current and former smokers and explore the associations between common genetic variants and PRISm status relative to control subjects.
- *Aim 3*: We hypothesize that subgroups exist within the PRISm cohort and explore the utility of unbiased machine learning approaches in identifying potentially pathobiologically distinct groups within PRISm.

## Materials and methods

### Study population

All subjects were participants in COPDGene (ClinicalTrials.gov Identifier NCT000608764); enrollment and exclusion criteria have been previously described [23]. COPDGene is a cross-sectional, observational study which enrolled self-identified non-Hispanic white (NHW) or African American (AA) current or former smokers aged 45–80 years with ≥10 pack-years of smoking. Institutional review board approval was obtained at each of the 21 participating clinical centers (please see Additional file 1– Additional Methods section for the names of the approving IRB offices); all subjects provided written informed consent. Subjects completed questionnaires, pre- and post-bronchodilator spirometry, 6-minute walk test, and volumetric chest computed tomography (CT) at full inspiration and expiration. All analyses were conducted using the COPDGene phenotype dataset released September 19, 2012.

### Variable definitions

**Figure 1:**
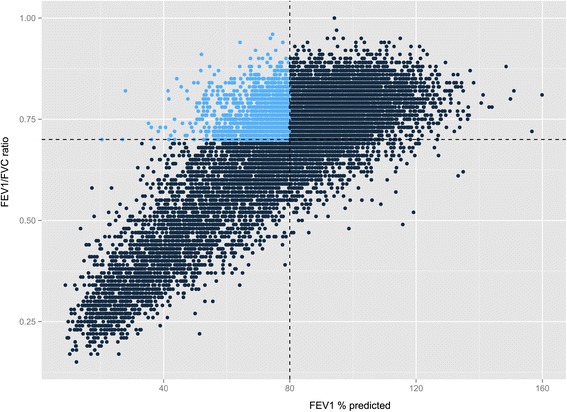
**Distribution of spirometry in COPDGene.** Legend: Forced expiratory volume in the first second (FEV_1_) is plotted on the x-axis while FEV_1_/FVC ratio is plotted on the y-axis. Dashed lines represent fixed-threshold criteria used to delineate Preserved Ratio Impaired Spirometry (PRISm) subjects (highlighted in blue-upper left quadrant), control (upper right quadrant), and COPD (lower left quadrant) subjects.

Percent predicted values and lower limits of normal (LLN) were calculated using post-bronchodilator spirometric values [24]. Fixed threshold-defined groups were as follows: PRISm subjects had an FEV_1_ < 80% predicted with an FEV_1_/FVC ≥ 0.7, control subjects had an FEV_1_ ≥ 80% with an FEV_1_/FVC ≥ 0.7, and COPD subjects had an FEV_1_ < 80% predicted with an FEV_1_/FVC < 0.7. The distribution of spirometry by FEV_1_ and FEV_1_/FVC in the COPDGene cohort is illustrated in Figure 1. LLN-defined cohorts were defined as follows: LLN-PRISm subjects had FEV_1_ < LLN with an FEV_1_/FVC ≥ LLN, LLN-controls had FEV_1_ ≥ LLN with an FEV_1_/FVC ≥ LLN, while LLN-COPD subjects had FEV_1_ < LLN and FEV_1_/FVC < LLN. The distribution of spirometry and delineation of LLN-defined populations are illustrated in Additional file 1: Figure S1. Additional variable definitions are available in detail (see Additional file 1).

### Aim 1: Epidemiological analysis

Univariate comparisons between PRISm subjects and control or COPD subjects were made using a Student’s t-test or Wilcoxon rank sum test for normal and non-normally distributed continuous variables, respectively, while Chi-square or Fisher’s exact tests were used for discrete variables. All nominally significant variables (p_univariate_ < 0.05), except lung function and medication use variables, were considered candidate independent variables for multivariate regression. Logistic regression using automated stepwise selection with binary PRISm status as the dependent variable was performed using SAS (v 9.3, Cary, NC); a significance level of 0.1 was specified for entry into the model and independent variables with a p-value <0.05 were retained in the final model. Additional details regarding stepwise selection are outlined in the Additional file 1 – Methods section. Non-significant candidate independent variables were tested as confounders and were retained if >10% change in the effect estimate was observed.

### Aim 2: Genetic analysis

Genome-wide single nucleotide polymorphism (SNP) genotyping data were obtained on the Illumina (San Diego, CA, USA) OmniExpress platform with additional genotypes imputed using MaCH [25] software and the 1000 Genomes [26] phased data (Hg19) for a total of ~14.9 million SNPs. Additional details regarding data processing and quality control are available (see Additional file 1). Genome-wide association testing for associations with binary PRISm status relative to control subjects was performed using an additive model, adjusted for age, sex, pack-years of smoking, BMI, current smoking status and principal components for genetic ancestry. Analyses were performed separately in non-Hispanic white and African American subjects using PLINK [27]; meta-analysis using standard error weighting was then performed using METAL software [28].

### Aim 3: Unsupervised cluster analysis to identify potential subgroups within PRISm

Unsupervised k-means clustering analyses were conducted on the subset of PRISm subjects (n_fixed threshold_ = 1,135 and n_LLN_ = 978) with complete data for six empirically chosen key input variables: TLC_CT_% predicted, FEV_1_% predicted, FEV_1_/FVC ratio, percent emphysema (%LAA-950_insp_), BMI, and segmental wall area percent. Normalized mutual information (NMI) using a five-fold cross-validation strategy was used to determine the optimal number of clusters [29]. To assess whether differences in the number or types of subgroups identified differed by race and to reduce the impact of population stratification, clustering and candidate gene testing were performed separately in NHW and AA subjects using R (2.15.0) [30]. Additional details regarding the cluster analysis are available in the Additional file 1.

## Results

### Aim 1: Epidemiological, functional, and radiographic predictors of PRISm status

Among the current and former smokers enrolled in the COPDGene cohort (n = 10,192), the mean age was 59.6 years, the mean pack-years smoked was 44.2, and mean body mass index (BMI) was 28.8; 53.5% of the cohort was male, 33.4% were African American, and 53.1% were current smokers. By fixed-threshold criteria, PRISm subjects account for 12.3% (n = 1,257) of the COPDGene cohort (Figure 1). Univariate comparisons between PRISm subjects and 1) control subjects and 2) COPD subjects are summarized in Table 1. PRISm subjects have the highest proportion of females and current smokers, increased mean body mass index (BMI) and decreased mean total lung capacity, as well as an increased prevalence of diabetes mellitus relative to both control and COPD subjects. PRISm subjects have significantly increased rates of respiratory medication use relative to smoking controls. However, within the PRISm cohort, the correlation between respiratory medication use and a history of physician-diagnosed asthma or evidence of bronchodilator responsiveness (BDR) on spirometry is poor (Additional file 1: Table S1); the simple kappa correlation between BDR and respiratory medication use ranged from 0.06 to 0.12. Thus, the majority of PRISm subjects with evidence of BDR do *not* report use of short- or long-acting beta agonists or inhaled steroids.

**Table 1:** **Characteristics of** ***P*** **reserved** ***R*** **atio** ***I*** **mpaired** ***S*** **piro** ***m*** **etry (PRISm), smoking control, and COPD subjects in COPDGene using fixed threshold criteria**

	**Control subjects**	**PRISm**	**COPD**
n	4388	1257	3690
Age	56.7 (8.4)*	57.2 (8.2)	63.4 (8.5)*
Sex (% Male)	52.9*	46.1	55.6*
African American	41.2	43.1	22.7*
Current smoker	59.7*	63.8	40.7*
Pack-years	37.2 (20.2)*	42.7 (24.2)	53.0 (27.5)*
Body Mass Index	28.9 (5.8)*	31.8 (7.3)	28.1 (6.3)*
FEV_1_% predicted	97.5 (11.5)*	70.2 (8.4)	50.2 (18.0)*
FVC% predicted	96.6 (11.9)*	71.5 (9.1)	76.3 (17.3)*
FEV_1_/FVC	0.79 (0.05)*	0.77 (0.05)	0.50 (0.13)*
Bronchodilator Responsiveness^†^	10.0*	13.7	36.6*
Total Lung Capacity_CT_% predicted	92.3 (14.6)*	79.9 (13.5)	101.5 (17.1)*
Segmental wall area percentage	60.1 (2.9)*	62.5 (3.1)	62.9 (3.1)*
Percent emphysema (% LAA-950_insp_)	2.0 (2.5)*	1.4 (2.5)	13.0 (12.8)*
Percent gas trapping (% LAA-856_exp_)	11.0 (9.7)	10.4 (9.1)	39.2 (20.8)*
Pi10	3.65 (0.11)*	3.73 (0.14)	3.72 (0.14)*
6 minute walk distance (feet)	1491.5 (350.7)*	1266.6 (366.9)	1174.8 (397.0)*
MMRC^‡^ Dyspnea score	0.8 (1.2)*	1.5 (1.5)	2.1 (1.4)*
Resting O_2_ saturation	97.1 (2.0)*	96.5 (2.5)	94.7 (3.6)*
Chronic bronchitis	12.6*	17.8	28.2*
Short acting beta-agonist use	12.0*	28.1	65.7*
Long acting beta-agonist use	4.4*	13.3	49.1*
Inhaled corticosteroid use	5.5*	16.4	51.2*
Oral corticosteroid use	0.5*	2	5.9*
Congestive heart failure	1.3*	4.6	5.4
Coronary artery disease	7.5*	13.5	16.5*
Diabetes mellitus	11.6*	21.6	13.1*
Hypertension	36.3*	49.1	50.6
Hyperlipidemia	34.3*	42.5	41.4
History of blood clot	2.8*	5.2	5.7
Peripheral vascular disease	1.3*	2.7	3.4
History of stroke	1.6*	3.5	3.6
Gastrointestinal reflux disease	20.3*	25.9	30.3*
History of compression fracture	3.4*	5.4	6.2
Currently employed	37.3*	28.9	25.3*
Physician-diagnosed asthma	11.4*	21.1	24.6*

Data are presented as mean (standard deviation) or percent. Preserved Ratio Impaired Spirometry (PRISm) defined as: FEV_1_/FVC ≥ 0.7 & FEV_1_ < 80% predicted. Control subjects defined as: FEV_1_/FVC ≥ 0.7 & FEV_1_ ≥ 80% predicted. Chronic Obstructive Pulmonary Disease (COPD) subjects defined as: FEV_1_/FVC < 0.7 & FEV_1_ < 80% predicted.

*Denotes univariate p-value < 0.05 when compared to PRISm subjects.

^†^Bronchodilator responsiveness considered present if the change in FEV_1_ or FVC was >200 mL *and* ≥ 12% predicted following administration of short acting inhaled beta-agonist.

^‡^MMRC = modified Medical Research Council.

Because controversy regarding the use of fixed thresholds to define respiratory impairment exists, we repeated the above analyses using lower limit of normal criteria to define the PRISm, control, and COPD groups. The prevalence of lower limit of normal-defined PRISm (LLN-PRISm) is 10.6% (n = 1,082) of the final cohort; characteristics of these subjects relative to LLN – control and LLN – COPD subjects are summarized in Additional file 1: Table S2. LLN-PRISm subjects continue to demonstrate the highest mean BMI and lowest mean TLC% predicted, as well as the highest prevalence of diabetes mellitus; however, the enrichment of female subjects is no longer present.

The overlap between fixed threshold-defined PRISm and LLN-PRISm is illustrated in Additional file 1: Figure S2; 883 subjects are consistently identified as PRISm by both criteria (simple kappa coefficient = 0.72). Subjects with PRISm by LLN criteria only (n = 199) are significantly older, have increased emphysema and gas trapping as well as a lower FEV_1_/FVC ratio relative to subjects identified as having PRISm by both fixed threshold and LLN criteria; 94.5% of these subjects (n = 188) have Stage 2 COPD by Global Initiative for Obstructive Lung Disease (GOLD)^11^ criteria. Of the subjects who have PRISm by fixed threshold criteria only (n = 374), 93.9% (n = 351) of these subjects are considered control subjects using LLN criteria. The reclassification of PRISm subjects by fixed-threshold and LLN-criteria is illustrated in Additional file 1: Figure S3.

Significant epidemiological predictors of PRISm status relative to control subjects in multivariate models for both fixed threshold and LLN-defined cohorts are presented in Table 2; none of the non-significant candidate independent variables were found to be confounders. The majority of risk factors identified were consistent regardless of whether fixed threshold or LLN criteria were used to define PRISm and control status and included increased cumulative pack-years, lower resting oxygen saturation, reduced 6-minute walk distance, increased MMRC dyspnea score, increased percent emphysema (after adjusting for TLC), decreased total lung capacity% predicted, increased segmental wall area percentage, and an increased prevalence of a history of peripheral vascular disease and physician-diagnosed asthma. Increased BMI and a history of diabetes mellitus were significant predictors only in the LLN cohort while female gender and increased age were significant only in the fixed threshold-defined cohort.

**Table 2:** **Significant predictors of** ***P*** **reserved** ***R*** **atio** ***I*** **mpaired** ***S*** **piro** ***m*** **etry (PRISm) status relative to control subjects in (a) fixed threshold-defined and (b) lower limit of normal (LLN)-defined cohorts in multivariate models**

**Panel (a) – significant predictors in fixed threshold- defined cohorts**		**Panel (b) – significant predictors in LLN defined cohorts**	
**Predictor**	**OR [95% CI]**	**Predictor**	**OR [95% CI]**
Pack-years	1.008 [1.005-1.012]	Pack-years	1.008 [1.004-1.012]
Resting oxygen saturation	0.907 [0.875-0.940]	Resting oxygen saturation	0.931 [0.897-0.966]
6 minute walk distance (per 100 feet)	0.948 [0.926-0.971]	6 minute walk distance (per 100 feet)	0.953 [0.929-0.977]
MMRC Dyspnea score	1.226 [1.153-1.304]	MMRC Dyspnea score	1.192 [1.116-1.273]
Percent emphysema	1.055 [1.020-1.091]	Percent emphysema	1.061 [1.029-1.094]
Total lung capacity% predicted	0.949 [0.943-0.955]	Total lung capacity% predicted	0.948 [0.942-0.954]
Segmental wall area percent	1.203 [1.168-1.238]	Segmental wall area percent	1.190 [1.154-1.227]
Peripheral vascular disease	1.777 [1.038-3.043]	Peripheral vascular disease	2.038 [1.254-3.314]
Physician-diagnosed asthma	1.391 [1.126-1.718]	Physician-diagnosed asthma	1.485 [1.193-1.848]
Age	1.025 [1.014-1.035]	Diabetes Mellitus	1.372 [1.111-1.695]
Sex (male)	0.705 [0.598-0.833]	African American race	0.266 [0.216-0.328]
Hyperlipidemia	1.216 [1.031-1.433]	Body mass index	1.026 [1.012-1.040]
		Current smoking	1.620 [1.337-1.962]
		Coronary artery disease	1.370 [1.002-1.874]

Variables tested but not retained in the final models:

Panel (a): body mass index, Pi10, current smoking, chronic bronchitis, bronchodilator responsiveness, congestive heart failure, coronary artery disease, diabetes, hypertension, history of blood clots, stroke, gastro-esophageal reflux disease, compression fractures, and current employment.

Panel (b): age, Pi10, chronic bronchitis, bronchodilator responsiveness, congestive heart failure, hypertension, hyperlipidemia, history of blood clots, stroke, gastro-esophageal reflux disease, compression fractures, and current employment.

No significant confounders (defined as causing >10% change in effect estimate) were found.

Multivariate models of PRISm status relative to both fixed-threshold and LLN-defined COPD are summarized in Additional file 1: Table S3. Analogous to the comparison of PRISm and control subjects, the majority of predictors identified on multivariate modeling of PRISm vs. COPD subjects were consistent regardless of whether fixed-threshold or LLN criteria were used. Increased BMI relative to COPD subjects was consistently identified as a predictor of PRISm status, as was decreased bronchodilator responsiveness. Radiographic differences, such as decreased measurements of emphysema, gas trapping, TLC, and segmental wall area thickness were also among the robustly identified predictors of PRISm relative to COPD subjects.

### Aim 2: Genetic associations between common genetic variants and PRISm status

During quality control of the genome-wide SNP genotyping data, six Klinefelter syndrome (47XXY) subjects were identified (Additional file 1: Figure S4). Five of the six Klinefelter subjects met criteria for PRISm by fixed threshold criteria while 3 met criteria for PRISm by LLN standards. This represents a significant enrichment of PRISm among Klinefelter syndrome subjects regardless of whether fixed threshold or LLN criteria were applied (Fisher’s exact p-values 1.53 x 10^−4^ and 0.02, respectively).

We performed a genome-wide association study (GWAS) of PRISm status relative to control subjects; results for the analysis in fixed threshold-defined cohorts are illustrated in Additional file 1: Figure S5. Although no genetic variant met the genome-wide threshold for significance (p-value < 5 x 10^−8^) in either the fixed threshold (Additional file 1: Table S4) or LLN – defined analyses (Additional file 1: Table S5), several SNPs with suggestive p-values were identified within the pleckstrin homology domain containing, family A member 5 (*PLEKHA5*) gene as well as within the voltage-dependent L-type calcium channel subunit beta-2 (*CACNB2*) gene. The most highly associated SNP from the fixed-threshold analysis (rs113840005 in *PLEKHA5*) was among the top 10 variants identified in the LLN-analysis. Considerable heterogeneity, as illustrated in the 10th – 90th percentile values for selected variables (Additional file 1: Table S6), exists among PRISm subjects and may contribute to the lack of genetic signal in GWAS analysis.

### Aim 3: Unsupervised cluster analysis to identify potential subgroups within PRISm subjects

**Figure 2:**
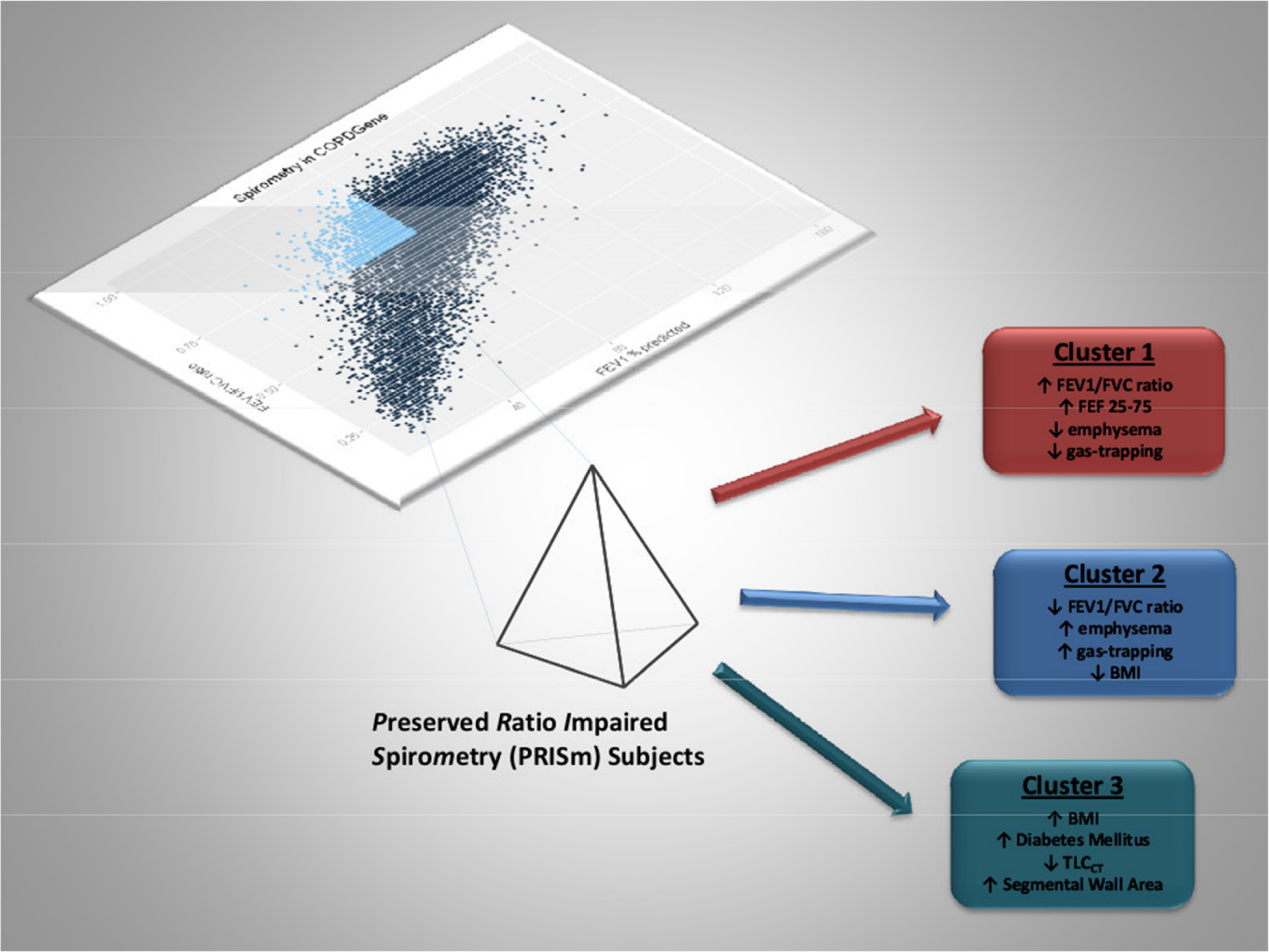
**Overview of cluster analysis in subjects with Preserved Ratio Impaired Spirometry (PRISm).**

**Figure 3:**
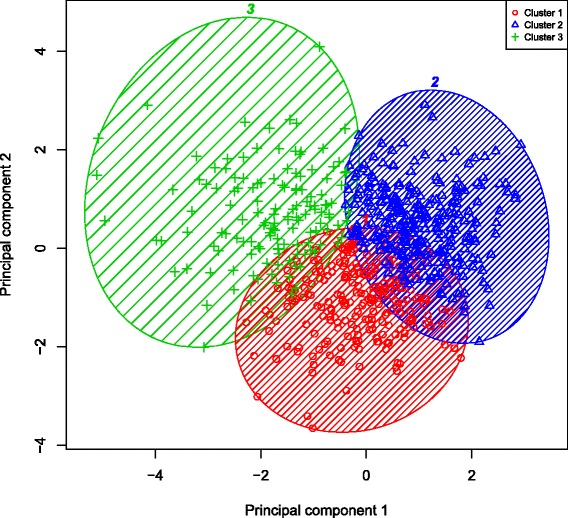
**Results of k-means clustering in fixed-threshold defined** ***P*** **reserved** ***R*** **atio** ***I*** **mpaired** ***S*** **pirometry (PRISm) in non-Hispanic whites.** Legend: Unsupervised k-means clustering was performed in non-Hispanic white subjects with PRISm. The first two principal components generated using the scaled 6 key input variables used for clustering (body mass index, FEV_1_%, predicted, FEV_1_/FVC ratio, percent emphysema, total lung capacity, and segmental wall area) are plotted on the x- and y-axes respectively.

**Figure 4:**
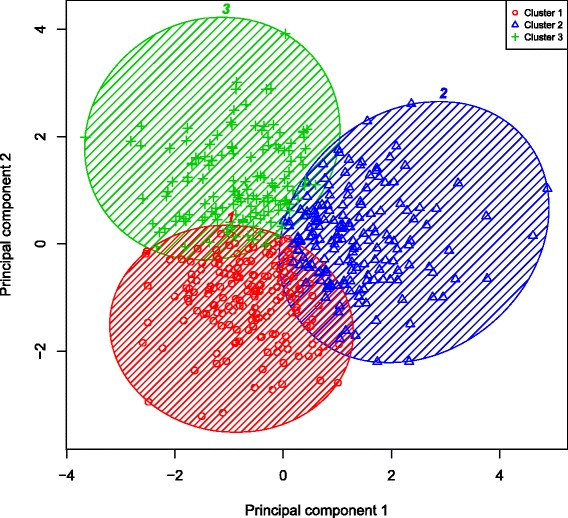
**Results of k-means clustering in fixed-threshold defined** ***P*** **reserved** ***R*** **atio** ***I*** **mpaired** ***S*** **pirometry (PRISm) in African Americans.** Legend: Unsupervised k-means clustering was performed in African American subjects with PRISm. The first two principal components generated using the scaled 6 key input variables used for clustering (body mass index, FEV_1_%, predicted, FEV_1_/FVC ratio, percent emphysema, total lung capacity, and segmental wall area) are plotted on the x- and y-axes respectively.

The subset of fixed threshold-defined PRISm subjects with complete data included in the k-means clustering analysis (n = 1,135) did not differ from the full cohort of PRISm subjects with respect to mean age, pack-years, BMI, or distribution by gender or current smoking status. Normalized mutual information (NMI) analysis using a five-fold cross-validation strategy demonstrated high cluster reproducibility for k = 3 clusters (Additional file 1: Table S7). An overview of the analysis is shown qualitatively in Figure 2 while the specific results of unsupervised k-means clustering in non-Hispanic white and African American PRISm subjects are illustrated in Figures 3 and 4, respectively. Clusters observed in NHW were reasonably reproducible in the AA, as illustrated in Additional file 1: Figure S6. Subgroup characteristics by cluster are summarized in Table 3. Members of Cluster 1 demonstrate the highest FEV_1_/FVC ratio and forced expiratory flow rate at 25%-75% of FVC (FEF_25–75_), as well as the lowest mean emphysema and gas trapping; we refer to this cluster as a putative “PRISm – Restricted cluster”. Members of Cluster 2 have the lowest mean BMI and FEV_1_/FVC ratio, as well as the highest mean emphysema and gas trapping; we refer to this cluster as the “PRISm – COPD cluster”. Finally, members of Cluster 3 have the highest mean BMI, the greatest degree of impairment in FEV_1_% predicted, the thickest segmental wall area, the lowest FEF_25–75_ flow rates, and the highest prevalence of diabetes mellitus and rates of bronchodilator responsiveness (BDR); we refer to this cluster as the “PRISm – Metabolic cluster”. Members of the Cluster 3 also demonstrate the highest mean MMRC dyspnea scores and the lowest mean 6 minute walk distance.

**Table 3:** **Results of unsupervised k-means clustering in (a) non-Hispanic white and (b) African American subjects with fixed threshold-defined** ***P*** **reserved** ***R*** **atio** ***I*** **mpaired** ***S*** **piro** ***m*** **etry (PRISm)**

	**(a) Non-Hispanic White subjects**			**(b) African American subjects**		
**Feature**	**Cluster 1**	**Cluster 2**	**Cluster 3**	**Cluster 1**	**Cluster 2**	**Cluster 3**
n	227	291	137	171	167	142
Age	57.9 (8.2)	62.2 (8.1)	59.2 (9.4)	52.9 (5.8)	54.6 (6.1)	52.6 (5.3)
Sex (% male)	34.8	45.7	51.1	47.4	56.9	45.1
Body Mass Index	30.9 (6.4)	30.4 (6.3)	36.2 (7.1)	32.0 (6.8)	27.3 (6.0)	35.7 (8.4)
Current smoker	53.3	43.6	49.6	84.8	83.8	80.3
Pack-years	42.8 (22.6)	46.6 (24.5)	50.9 (29.7)	35.6 (18.5)	39.6 (24.0)	38.1 (21.4)
FEV_1_% predicted	74.2 (4.3)	73.4 (5.3)	60.5 (7.8)	73.3 (5.1)	72.7 (6.2)	62.5 (8.9)
FEV_1_/FVC	0.80 (0.04)	0.74 (0.03)	0.74 (0.04)	0.83 (0.04)	0.74 (0.03)	0.75 (0.03)
FEF _25–75_	2.21 (0.65)	1.58 (0.47)	1.41 (0.47)	2.38 (0.71)	1.58 (0.50)	1.42 (0.43)
Resting O2 saturation	96.4(2.3)	96.3 (2.1)	95.2 (2.9)	97.1 (2.2)	97.1 (2.3)	96.6 (2.7)
TLC_CT_^*^% predicted	79.4 (9.8)	90.4 (10.7)	74.4 (12.7)	71.0 (11.1)	83.6 (12.3)	71.6 (12.3)
Percent emphysema	0.6 (0.7)	2.6 (2.7)	0.9 (1.1)	0.5 (0.6)	2.7 (4.5)	0.8 (1.0)
Segmental Wall Area%	62.0 (2.5)	60.6 (2.3)	64.7 (2.6)	62.4 (2.8)	61.9 (2.9)	65.7 (2.5)
Percent Gas Trapping	6.5 (6.0)	13.5 (8.6)	9.8 (7.1)	7.2 (7.3)	14.8 (13.5)	9.4 (7.5)
Diabetes Mellitus	18.5	14.4	36.5	22.8	15.6	27.5
Hypertension	36.6	47.4	56.2	53.8	51.5	54.2
Hyperlipidemia	45.4	58.8	59.9	31.0	24.0	29.6
Chronic bronchitis	16.3	15.8	32.9	11.1	19.2	14.8
Bronchodilator Response^†^	11.5	13.5	22.2	11.4	9.2	20.1
MMRC^‡^	1.154 (1.32)	1.13 (1.29)	1.95 (1.50)	1.63 (1.53)	1.57 (1.47)	1.96 (1.53)
6 minute walk distance	1386.5 (347.1)	1392.2 (320.4)	1132.6 (390.2)	1219.9 (349.4)	1279.5 (338.1)	1099.2 (342.1)

Results are reported as mean (SD) or percent.

Preserved Ratio Impaired Spirometry (PRISm) defined as: FEV_1_/FVC ≥ 0.7 & FEV_1_ < 80% predicted.

^*^TLC_CT_ = Total Lung Capacity by computed tomography.

^†^Bronchodilator response considered present if the change in FEV_1_ or FVC was >200 mL *and* ≥ 12% predicted following administration of short acting inhaled beta-agonist.

^‡^MMRC = Modified Medical Research Council.

We also performed cluster analysis on the LLN-defined PRISm cohort; the subset of subjects with complete data (n = 978) did not differ from the full LLN-PRISm cohort with respect to mean age, pack-years smoked, BMI, or distribution by gender; there were significantly fewer current smokers (60% vs. 70.2%) in the subset with complete data used for cluster analysis. NMI and silhouette width analysis demonstrated high cluster reproducibility for k = 4 clusters (Additional file 1: Table S7). The results of unsupervised k-means clustering in NHW and AA subjects are illustrated in Additional file 1: Figure S7 (panels (a) and (b) respectively). Separation between clusters in each of these analyses (NHW and AA) is not as distinct as in the fixed threshold analysis; additionally, the clusters found in NHW did not appear to overlap well with clusters identified in the AA analysis (Additional file 1: Figure S8). Characteristics of each cluster are summarized in Additional file 1: Table S9. We have putatively assigned Clusters 1, 2, and 3 to be analogous to the “PRISm-restrictive”, “PRISm-COPD”, and “PRISm-metabolic” subtypes described in the fixed-threshold analysis. Members of Cluster 4 have the highest rates of current smoking; however, beyond that, the clusters appear to represent relatively distinct subgroups in NHW and AA subjects. In the NHW LLN-PRISm Cluster 4, subjects have the lowest BMI and highest resting oxygen saturation and best exercise capacity while Cluster 4 subjects in the AA LLN-PRISm analysis appear to be more ill with the greatest impairment in FEV1% predicted, increased segmental wall area thickness and decreased FEF_25–75_.

Genetic variants previously described in studies of COPD, interstitial lung disease, and metabolic phenotypes were examined for associations with the clusters identified in the fixed-threshold analysis (Additional file 1: Table S10). The minor (risk) allele frequency of rs8050136, located in the first intron of the fat mass and obesity associated (*FTO*) gene, by subgroup is illustrated in Additional file 1: Figure S9; a relative enrichment of risk alleles in the PRISm – Metabolic subgroup was noted among African American subjects (ANOVA p-value 0.05), however, this enrichment was not statistically significant among non-Hispanic white subjects.

## Discussion

In this manuscript, we examine detailed demographic, spirometric, and radiographic features of subjects with *P*reserved *R*atio *I*mpaired *S*pirometry and leverage these data to explore genetic associations and subgroups within the cohort. We confirm the overall prevalence of PRISm within current and former smokers in our cohort is consistent with the prevalence reported in other cross-sectional studies, including several population-based studies [10–13,15,31]. We affirm previously reported associations with body mass index and diabetes mellitus and report novel associations with radiographic and functional predictors of PRISm status (Aim 1). While no genome-wide significant genetic predictors were identified in our GWAS studies, we uncovered a novel association between PRISm and Klinefelter’s syndrome (Aim 2). Finally, the results of unsupervised clustering analysis demonstrate 3 clusters which may represent pathobiologically distinct subgroups within the PRISm cohort.

### Aim 1: Epidemiology of PRISm

As with obstructive lung diseases, controversy regarding the delineation between normal and abnormal exists for PRISm. Differences in the populations defined by fixed threshold (i.e. GOLD criteria) versus lower limit of normal FEV_1_ criteria likely contribute to differences in associations with certain epidemiological predictors identified in our analysis; for example, the enrichment of African Americans in the LLN-defined PRISm cohort may reflect less accurate population-based prediction equations for or increased variability in African Americans rather than a distinct pathobiological process.

Despite the lack of a consensus definition for PRISm, the majority of associations reported in our study were remarkably consistent regardless of whether fixed (GOLD) or LLN criteria were utilized. Previously reported associations with increased mean BMI and a high prevalence of comorbid conditions such as diabetes mellitus [6,9,10,12,13,15,18] were observed in our cohort on univariate analyses. We additionally confirm associations with decreased total lung capacity and decreased emphysema first reported in our analysis of PRISm subjects among the first 2500 subjects recruited in COPDGene [8].

In multivariable models, PRISm subjects had increased cumulative exposure to tobacco smoke as well as an increased prevalence of physician-diagnosed asthma and peripheral vascular disease relative to control subjects. These factors may contribute to the increase in symptoms as assessed through the MMRC score, decreased exercise tolerance, and decreased resting oxygen saturation also observed in this cohort relative to control subjects. In multivariate models comparing PRISm with COPD subjects, increased body mass index and an increased prevalence of diabetes mellitus were consistently identified as predictors; whether these factors are pathobiologically related to the development of these two distinct disease states is a topic that warrants investigation in the future. Radiographic variables, such as percent emphysema and TLC, were among the most consistently identified predictors of PRISm status in multivariate models relative to *both* control and COPD subjects.

The role of increased BMI among PRISm subjects continues to deserve special consideration. Although obesity has been associated with proportionate decreases in FEV_1_ and FVC as well as decreases in TLC, lung function values of obese subjects typically remain within the normal range [32]; thus the degree of impairment in lung function in PRISm subjects is unlikely to be due solely to the mechanical properties of increased body mass. This supposition is supported by the divergent trends in the prevalence of overweight and obesity relative to PRISm over the last half century (Additional file 1: Figure S10); the dramatic increase in the prevalence of overweight and obesity [33] is not reflected in the relatively stable prevalence of PRISm [7–9,11–13,15,16,22,31,34].

### Aim 2: Genetic associations

The association between Klinefelter’s syndrome and PRISm is consistent with previous reports of an increased prevalence of restriction [35–37] in this population; in fact, all six Klinefelter subjects identified demonstrated a TLC_CT_ < 80% predicted (data not shown). We acknowledge the limitations associated with the use of male prediction equations for lung function in this subgroup, as traditional formulas do not account for the eunuchoid proportions which characterize this syndrome. However, given previous reports of decreased lung compliance [36], an increased prevalence of respiratory symptoms [38,39], and increased mortality due to respiratory causes among Klinefelter’s subjects [40,41], we believe the association may be indicative of true pathobiological differences and warrants additional investigation in the future.

Although no single genetic variant met the accepted genome-wide threshold for significance, we identified suggestive associations between PRISm and variants within the *PLEKHA5* and *CACNB2* genes. rs113840005, which was a top variant in both the fixed threshold and LLN analyses, is located within an intron of the *PLEKHA5* gene. Multiple splice variants of this gene have been identified; some isoforms are ubiquitously expressed while other isoforms are highly specific to tissue type and developmental stage. In general, the protein products of the *PLEKHA5* gene are typically located in the cytosol of cells and are believed to contribute to intracellular signaling and cytoskeletal organization [42]; the mechanism by which variants in this gene are associated with PRISm is unclear. Intronic variants within the *CACNB2* gene were among the most strongly associated in the fixed-threshold analysis. Analogous to *PLEKHA5*, multiple isoforms of the protein product exist. Variants within this gene have been associated with blood pressure levels and hypertension [43,44] as well as Brugada syndrome [45–47]. Additional investigations into the mechanism behind the association of variants at this locus with PRISm are warranted.

### Aim 3: Subgroup identification

Clinical management strategies for PRISm are poorly defined and reflect the low specificity of spirometric measurements alone in identifying distinct disease processes in this cohort [13,14]. Overt and subclinical interstitial lung disease [5], chest wall abnormalities, neuromuscular and functional impairments, as well as airway diseases (such as asthma and chronic obstructive pulmonary disease) which have classically been associated with obstruction [6,9,48], can all produce the PRISm pattern on spirometry; what remains unknown is the proportion attributable to each process and how to identify different groups of subjects. When we incorporated key clinical and radiographic variables with existing spirometric data into an unbiased clustering algorithm, we were able to identify clinically relevant subtypes within the PRISm cohort.

Previous studies have supported the existence of an airway disease/COPD subgroup among PRISm subjects [6,9]; we were likewise able to identify a putative COPD subgroup (Cluster 2 in both the NHW and AA analyses) with evidence of relatively increased emphysema and gas trapping, preserved TLC, and a relatively reduced FEV_1_/FVC ratio. Interestingly, this group appears to have the lowest degree of physiological impairment; they experience the least dyspnea (as assessed by MMRC score), have the best exercise capacity (highest 6MWD), and the least hypoxemia. In a longitudinal study by Guerra et al. [9], approximately one-third of subjects with PRISm eventually developed airflow obstruction on spirometry – Cluster 2 may be enriched for subjects with “early COPD” who have not yet developed the classical obstructive pattern. Longitudinal data, which is currently being collected in the COPDGene cohort, will be crucial to investigating this hypothesis.

The PRISm-metabolic subgroup represents a highly symptomatic and functionally limited group for whom treatment options have not been systematically explored. This subgroup has the greatest degree of spirometric impairment in FEV_1_ which may be related to the increased subsegmental airway wall thickness. Given these findings, as well as the high prevalence of bronchodilator responsiveness in this subgroup, the benefit of inhaled steroids and/or bronchodilators in this subgroup presents a clinically relevant question for future studies.

In summary, we have analyzed the epidemiological and radiographic predictors, explored clinically relevant putative subgroups, and identified a novel association with Klinefelter’s syndrome in PRISm. The strengths of the current study include the utilization of a large cohort with rich data in multiple domains as well as the application of rigorous, unbiased interrogations to both characterize and subtype this relatively understudied syndrome. Despite this, we acknowledge the following limitations. First, the lack of visual assessments of CT data for the majority of the cohort limits our ability to ascertain the impact of chest wall or diaphragmatic abnormalities and atypical interstitial/parenchymal infiltrates among the PRISm cohort. Second, although this cohort is the largest to date with genetic data available, the number of subjects is modest in the context of genome-wide association studies and limits our power to detect variants of modest effect sizes. Lastly, the degree to which the findings reported in our study are generalizable to other populations, such as non-smokers and subjects outside of the United States, should be explored. Future work in independent populations of PRISm subjects, as well as *in vivo* and *in vitro* work in model systems, to explore the biological mechanisms behind the associations reported in our manuscript are warranted.

## Additional file

Additional file 1
**Additional Methods - Additional information on cohorts, variables, and analyses.** **Table S1** - Respiratory medication use by physician diagnosed asthma and BDR, **Table S2**-Characteristics of LLN-defined cohorts, **Table S3** - Multivariate predictors of LLN-PRISm, **Table S4**-Top 10 associations of Fixed-threshold PRISm vs. Controls GWAS, **Table S5**-Top 10 associations of LLN-PRISm vs. Controls GWAS, **Table S6**- 10th-90th percentiles of variables in PRISm, **Table S7**-Normalized mutual information and silhouette width for fixed-threshold PRISm cluster analysis, **Table S8** - Normalized mutual information and silhouette width for LLN-PRISm cluster analysis, **Table S9**-Results of unsupervised k-means clustering in LLN-PRISm, **Table S10**-Candidate gene testing in fixed-threshold PRISm subgroups. **Figure S1** - Distribution of spirometry by LLN criteria, **Figure S2** - Overlap between fixed-threshold and LLN PRISm, **Figure S3** - Reclassification of PRISm subjects by fixed-threshold and LLN criteria, **Figure S4** - Identification of Klinefelter subjects by X and Y chromosome intensity analysis, **Figure S5** - QQ and Manhattan plots of fixed-threshold PRISm GWAS, **Figure S6** - Superimposed K-means clustering results in NHW+AA subjects (fixed-threshold PRISm), **Figure S7** - K-means clustering results in LLN-PRISm, **Figure S8 -** Superimposed K-means clustering results in NHW + AA, **Figure S9** - MAF of rs8050136 by fixed-threshold PRISm subgroup, **Figure S10** - Prevalence of overweight/obesity and PRISm (1960-2010).

## Abbreviations

PRISm: Preserved ratio impaired spirometry
FEF_25–75_: Forced expiratory flow rate at 25%-75% of forced vital capacity
FEV1: Forced expiratory volume in the first second
FVC: Forced vital capacity
GWAS: Genome wide association study
GOLD: Global initiative for obstructive lung disease
%LAA-950_insp_: Percent low attenuation areas at −950 Hounsfield units on inspiratory CT scan
LLN: Lower limits of normal
BMI: Body mass index
NHW: Non-Hispanic white
NMI: Normalized mutual information
AA: African American
CT: Computed tomography
COPD: Chronic obstructive pulmonary disease
SNP: Single nucleotide polymorphism
BDR: Bronchodilator responsiveness
MMRC: Modified Medical Research Council
TLC: Total lung capacity
