# Supplementary material for: Epidemiology, genetics, and subtyping of preserved ratio impaired spirometry (PRISm) in COPDGene
Source: Respir Res. 2014 Aug 6;15(1):89. doi: 10.1186/s12931-014-0089-y (PMC4256936; doi:10.1186/s12931-014-0089-y)
Supplement: Additional file 1 — Additional Methods - Additional information on cohorts, variables, and analyses. Table S1 - Respiratory medication use by physician diagnosed asthma and BDR, Table S2-Characteristics of LLN-defined cohorts, Table S3 - Multivariate predictors of LLN-PRISm, Table S4-Top 10 associations of Fixed-threshold PRISm vs. Controls GWAS, Table S5-Top 10 associations of LLN-PRISm vs. Controls GWAS, Table S6- 10th-90th percentiles of variables in PRISm, Table S7-Normalized mutual information and silhouette width for fixed-threshold PRISm cluster analysis, Table S8 - Normalized mutual information and silhouette width for LLN-PRISm cluster analysis, Table S9-Results of unsupervised k-means clustering in LLN-PRISm, Table S10-Candidate gene testing in fixed-threshold PRISm subgroups. Figure S1 - Distribution of spirometry by LLN criteria, Figure S2 - Overlap between fixed-threshold and LLN PRISm, Figure S3 - Reclassification of PRISm subjects by fixed-threshold and LLN criteria, Figure S4 - Identification of Klinefelter subjects by X and Y chromosome intensity analysis, Figure S5 - QQ and Manhattan plots of fixed-threshold PRISm GWAS, Figure S6 - Superimposed K-means clustering results in NHW+AA subjects (fixed-threshold PRISm), Figure S7 - K-means clustering results in LLN-PRISm, Figure S8 - Superimposed K-means clustering results in NHW + AA, Figure S9 - MAF of rs8050136 by fixed-threshold PRISm subgroup, Figure S10 - Prevalence of overweight/obesity and PRISm (1960-2010). [file 12931_2014_89_MOESM1_ESM.pdf]

Epidemiology, Genetics, and Subtyping of Subjects with Preserved Ratio Impaired Spirometry (PRISm) in COPDGene

Additional Files

Emily S. Wan, MD; Peter J. Castaldi, MD; Michael H. Cho, MD; John E. Hokanson, PhD; Elizabeth A. Regan, MD, PhD; Barry J. Make, MD; Terri H. Beaty, PhD; MeiLan K. Han, MD; Jeffrey L. Curtis, MD; Douglas Curran-Everett, PhD; David A. Lynch, MD; Dawn L. DeMeo, MD; James D. Crapo, MD; Edwin K. Silverman, MD, PhD; and the COPDGene Investigators

Additional Methods

Study Population

Race and ethnicity data were collected as part of the COPDGene Study. Subjects were asked to self-identify their race and ethnicity based on the investigator-defined categories of non-Hispanic white and African American. These data were necessary for the analysis of genetic risk factors for chronic respiratory diseases. The following institutional review boards (IRB) approved the COPDGene study: Partners IRB, Ann Arbor VA Research IRB, Baylor University IRB, Columbia University Medical Center IRB, Duke University Health System IRB, Health Partners IRB, Johns Hopkins Medicine IRB, Harbor UCLA Medical Center IRB, Michael E. DeBakey VA Medical Center IRB, Minneapolis VA Health Care System IRB, Morehouse School of Medicine IRB, National Jewish Health IRB, Temple University IRB, University of Alabama IRB, University of California San Diego Human Research Protections Program, University of Iowa IRB, University of Michigan Medical School IRB, University of Minnesota IRB, University of Pittsburgh IRB, Office of the IRB University of Texas Health Science Center San Antonio.

Variable definitions

Medication lists were constructed during the subject interview, and then classes of respiratory medications were designated by the research coordinator. For this analysis, we focused on short-acting beta-agonists (SABA), long-acting beta agonists (LABA), inhaled corticosteroids (ICS), and oral corticosteroids. Bronchodilator responsiveness was considered present if the change in FEV_1 or FVC was ≥200 mL and ≥12% change from baseline following administration of a short-acting inhaled beta-agonist. Chronic bronchitis was considered present if subjects reported a cough productive of phlegm on most days for 3 consecutive months for at least 2 years. Comorbid conditions were assessed by self-report on a standardized questionnaire. Coronary artery disease was considered present if the subject reported a history of coronary artery disease, coronary artery bypass grafting, angioplasty, heart attack, or angina.

Total lung capacity (TLC_{CT}) in liters was calculated from volumetric CT measurements at full inspiration; percent predicted values were calculated from published references[1, 2]. Percent emphysema was calculated as the percentage of voxels within the lung with an attenuation < -950 Hounsfield units at full inspiration ($\%LAA\text{-}950_{insp}$). Gas trapping was defined as the percentage of lung with an attention less than -856 HU on expiratory chest computed tomography. Segmental wall area was determined from quantitative airway measurements made at the fourth generation bronchi. Pi10 is a standardized variable which describes the wall thickness of a hypothetical airway with an internal perimeter of 10mm; the value is extrapolated by plotting the square root of multiple airway wall measurements against the internal perimeter of the airway[3].

Epidemiological analysis

Stepwise selection as implemented in SAS (v 9.3, Cary, NC) was used determine significant variables in the multivariate regressions[4]. User-specified significance levels for entry into and retention in the model were 0.1 and 0.05, respectively. For stepwise regression, an intercept-only model is computed and score statistics are assigned to each candidate variable. The variable with the most significant score meeting the threshold for entry into the model is added first; if the candidate variable remains significant (i.e. – less than the user-specified level for retention in the model), it is not removed and the next most significant candidate variable is entered in to the model. This is performed iteratively until no additional candidate variables meet the significance level for entry into the model. Variables not retained in the final multivariate model were reintroduced into the final model and the impact on the effect estimate (i.e. odds ratio) of significant (retained) variables was ascertained; non-significant variables which caused a +/- 10% change in the effect estimate of significant variables were considered confounders and were retained in the final model.

Genetic data processing

Quality control and pre-processing of genetic data by sample and by probe in this cohort have been previously described[5]. Gender checking was performed by examining the mean intensity of SNPs on the X and Y chromosomes against the self-reported gender from questionnaire data.

Cluster analysis

For the unsupervised k-means clustering analysis, we used percent predicted values for total lung capacity derived using published references[1, 2] and log transformed percent emphysema at -950 HU + 1. All input variables were normalized prior to clustering; clustering was performed using the *kmeans* function in R (release 2.15.0)[6] using the default method of Hartigan and Wong[7], 50 starting centers, and 10 maximum iterations. In k-means clustering, the number of clusters is fixed by the user. An initial set of aggregation centers, or seeds, are determined and a distance measure (often Euclidean distance) for each data point to each of the centers is computed. Each data point is assigned to the nearest seed and the cluster center is recomputed. Each data point is then reassigned by distance measure to the new cluster centers. This process is repeated iteratively until no reclassification is necessary and clusters are presumed stable.

Additional Results

<u>Additional File - Table S1</u> – Rates of respiratory medication use by history of physician-diagnosed asthma and bronchodilator responsiveness* among fixed-threshold defined *P*reserved *R*atio *I*mpaired *S*piro*m*etry (PRISm) subjects (n_{total} = 1,257)

	Percent of subjects reporting medication use with concurrent:	
Medication	Physician diagnosed asthma	Bronchodilator responsiveness†
Inhaled corticosteroids	53.4%	20.6%
Short-acting beta agonists	51.3%	21.8%
Long-acting beta agonists	49.1%	18.7%

*Bronchodilator responsiveness defined as an increase ≥200mL *and* >12% in FEV_1 or FVC following administration of short acting bronchodilator

† n = 1240 (17 subjects missing bronchodilator response value)

<u>Additional File – Table S2</u> – Characteristics of subjects with Lower Limit of Normal (LLN)-defined Preserved Ratio Impaired Spirometry (PRISm), control subjects, and obstructive spirometry in COPDGene

	LLN controls	LLN PRISm	LLN COPD
n	5095	1082	3315
Age	57.6 (8.8)	58.1 (8.4)	62.9 (8.6) *
Sex (% Male)	53.0	55.0	55.1
African American	40.0*	31.7	22.9*
Current smoker	57.9	61.0	41.4*
Pack-years	38.2 (21.1)*	45.8 (26.4)	52.9 (27.3) *
Body Mass Index	28.9 (5.8) *	32.1 (7.3)	27.9 (6.3) *
FEV_1 % predicted	95.5 (12.3) *	67.0 (8.2)	47.3 (17.0) *
FVC % predicted	95.5 (12.5) *	69.5 (9.0)	75.1 (17.3) *
FEV_1/FVC	0.78 (0.06) *	0.75 (0.06)	0.48 (0.13) *
Bronchodilator Responsiveness†	10.7*	14.4	37.8*
Total Lung Capacity % predicted	92.0 (14.8) *	80.3 (13.7)	102.5 (17.1) *
Segmental wall area percentage	60.2 (2.9) *	62.5 (3.0)	63.1 (3.1) *
Percent emphysema (% $LAA\text{-}950_{insp}$)	2.2 (2.8) *	1.8 (2.9)	13.9 (13.0) *
Percent gas trapping (% $LAA\text{-}856_{exp}$)	11.7 (10.1)	12.0 (10.1)	41.0 (20.8) *
Pi10	3.65 (0.11) *	3.72 (0.14)	3.73 (0.14)
6 minute walk distance (feet)	1475.3 (357.8) *	1274.3 (368.7)	1157.2 (396.8) *
MMRC‡ Dyspnea score	0.8 (1.2) *	1.5 (1.5)	2.2 (1.4) *
Resting O2 saturation	97.0 (2.1) *	96.3 (2.4)	94.5 (3.7) *
Chronic bronchitis	12.7 *	19.7	29.3 *
Short acting beta-agonist use	13.1*	31.0	69.0*
Long acting beta-agonist use	5.2*	16.2	51.9*
Inhaled corticosteroid use	6.4*	19.3	54.2*
Oral corticosteroid use	0.5*	2.0	6.3*
Congestive heart failure	1.5*	5.5	5.4
Coronary artery disease	8.3*	16.5	16.1
Diabetes mellitus	12.0*	24.4	12.3*
Hypertension	38.3*	49.0	49.4
Hyperlipidemia	36.0*	45.8	40.2*
History of blood clot	3.2*	6.0	5.3
Peripheral vascular disease	1.4*	4.0	3.3
History of stroke	1.8*	3.8	3.4
Gastrointestinal reflux disease	21.2*	27.0	30.0
History of compression fracture	3.4*	5.9	6.5
Currently employed	36.3*	29.3	24.8*
Physician-diagnosed asthma	11.7*	21.0	25.3*

Data are presented as mean (standard deviation) or percent.
Preserved Ratio Impaired Spirometry (PRISm) defined as: $FEV_1/FVC \geq 0.7$ & $FEV_1 < 80\%$ predicted
Control subjects defined as: $FEV_1/FVC \geq 0.7$ & $FEV_1 \geq 80\%$ predicted
Chronic Obstructive Pulmonary Disease (COPD) subjects defined as: $FEV_1/FVC < 0.7$ & $FEV_1 < 80\%$ predicted
* Denotes univariate $p<0.05$ when compared to LLN-PRISm subjects.
†Bronchodilator responsiveness considered present if the change in FEV_1 or FVC was >200mL *and* ≥ 12% predicted following administration of short acting inhaled beta-agonist.
‡ MMRC = modified Medical Research Council

Additional File – Table S3 – Significant predictors of *P*reserved *R*atio *I*mpaired *S*pirometry (PRISm) status relative to subjects with chronic obstructive pulmonary disease in (a) fixed threshold-defined and (b) lower limit of normal (LLN)-defined cohorts in multivariate models

Panel (a) – Significant predictors in fixed threshold- defined cohorts		Panel (b) – Significant predictors in LLN defined cohorts	
Predictor	OR [95% CI]	Predictor	OR [95% CI]
Body mass index	1.046 [1.028-1.063]	Body mass index	1.059 [1.040-1.079]
Resting oxygen saturation	1.096 [1.049-1.145]	Resting oxygen saturation	1.139 [1.086-1.195]
MMRC Dyspnea score	0.893 [0.825-0.967]	MMRC Dyspnea score	0.809 [0.745-0.877]
Percent gas trapping	0.938 [0.926-0.951]	Percent gas trapping	0.948 [0.936-0.960]
Percent emphysema	0.883 [0.841-0.927]	Percent emphysema	0.885 [0.848-0.924]
Total lung capacity % predicted	0.946 [0.938-0.954]	Total lung capacity % predicted	0.945 [0.937-0.953]
Segmental wall area percent	0.865 [0.832-0.898]	Segmental wall area percent	0.853 [0.821-0.887]
Bronchodilator responsive	0.352 [0.272-0.456]	Bronchodilator responsive	0.342 [0.261-0.447]
Diabetes Mellitus	1.459 [1.103-1.931]	Diabetes Mellitus	1.971 [1.473-2.637]
Age	0.963 [0.950-0.976]	African American race	0.691 [0.527-0.905]
Sex (male)	0.506 [0.405-0.633]		
Chronic bronchitis	0.762 [0.587-0.989]		

Variables tested but not retained in the final models:
Panel (a): African American race, current smoking, pack-years smoked, Pi10, six minute walk distance, coronary artery disease, gastro-esophageal reflux disease, physician-diagnosed asthma, and current employment.
Panel (b): age, current smoking, pack-years smoked, six minute walk distance, chronic bronchitis, hyperlipidemia, physician-diagnosed asthma, and current employment.
No significant confounders (defined as causing >10% change in effect estimate) were found.

<u>Additional File – Table S4</u> - Top 10 results of GWAS meta-analysis of fixed threshold-defined *P*reserved *R*atio *I*mpaired *S*pirometry (PRISm) subjects versus control subjects

Marker	Chromosome	Nearest Gene	p-value
rs113840005	12	*PLEKHA5*	1.80E-07
rs7722385	5	*MYO10*	2.22E-07
rs112374690	8	*PVT1*	2.54E-07
rs12246467	10	*CACNB2*	3.11E-07
rs77442831	10	*CACNB2*	3.72E-07
rs12252650	10	*CACNB2*	5.22E-07
rs9663417	10	*CACNB2*	5.24E-07
rs7722821	5	*MYO10*	6.34E-07
rs73333595	12	*PLEKHA5*	6.60E-07
rs4731411	7	*MIR129-1*	7.35E-07

Individual genome-wide association analyses, adjusting for age, sex, pack-years, body mass index, current smoking status, and principal components for genetic ancestry, were conducted separately in non-Hispanic white (n = 698 cases / 2534 controls) and African American (n = 518 cases / 1749 controls) subjects. Meta-analysis was performed using standard error weighting.

<u>Additional File – Table S5</u> – Top 10 results of GWAS meta-analysis of lower limit of normal defined Preserved Ratio Impaired Spirometry (LLN-PRISm) subjects versus control subjects

Marker	Chromosome	Nearest Gene	p-value
rs114686219	3	*ZCWPW2*	4.32E-07
rs183231082	10	*FAM13C*	4.77E-07
rs113840005	12	*PLEKHA5*	8.82E-07
rs139923689	11	*CNTN5*	9.18E-07
rs181659752	3	*ZIC4*	9.27E-07
rs140202167	8	*DECR1*	9.33E-07
rs183026977	10	*SFTA1P*	9.35E-07
rs6140864	20	*PLCB4*	9.63E-07
rs1568903	4		1.17E-06
rs13121426	4		1.39E-06

Individual genome-wide association analyses, adjusting for age, sex, pack-years, body mass index, current smoking status, and principal components for genetic ancestry, were conducted separately in non-Hispanic white (n = 723 cases / 3006 controls) and African American (n = 330 cases / 1968 controls) subjects. Meta-analysis was performed using standard error weighting.

<u>Additional File – Table S6</u> – 10th and 90th percentile values of selected variables in subjects with *P*reserved *R*atio *I*mpaired *S*piro*m*etry (PRISm) as defined by (a) fixed threshold and (b) lower limit of normal (LLN) criteria

GOLD - PRISm	Variable	LLN - PRISm
22.9 – 41.8	BMI	23.3 – 42.3
58.5 – 78.7	FEV_1 (% predicted)	55.7 – 75.9
0.1 – 3.5	Emphysema (%)	0.1 – 4.4
62.5 – 97.2	Total lung $capacity_{CT}$ (% predicted)	62.9 – 97.8

<u>Additional File – Table S7</u>– Normalized mutual information and silhouette widths derived from five-fold cross validation analysis in (a) non-Hispanic white (n = 655) and (b) African American (n = 480) subjects with fixed threshold-defined Preserved Ratio Impaired Spirometry (PRISm)

(a) Results for non-Hispanic white fixed-threshold PRISm subjects

	Normalized Mutual Information		Silhouette Width	
k =	mean	SD	mean	SD
2	0.7297	0.0953	0.1905	0.0207
3	0.8143	0.1072	0.1591	0.0109
4	0.8992	0.0624	0.1531	0.0124
5	0.7591	0.1289	0.1232	0.0062
6	0.6676	0.0904	0.1219	0.0119
7	0.6860	0.0735	0.1219	0.0155

(b) Results for African American fixed-thresold PRISm subjects

	Normalized Mutual Information		Silhouette Width	
k	mean	SD	mean	SD
2	0.7551	0.0982	0.1586	0.0184
3	0.8827	0.0431	0.1699	0.0124
4	0.7165	0.1200	0.1494	0.0208
5	0.8290	0.1250	0.1512	0.0216
6	0.7558	0.0870	0.1369	0.0101
7	0.6967	0.0782	0.119	0.0141

Additional File – Table S8 – Normalized mutual information and silhouette widths derived from five-fold cross validation in (a) non-Hispanic white (n = 681) and (b) African American (n = 297) subjects with lower limit of normal-defined Preserved Ratio Impaired Spirometry (PRISm).

(a) Results for non-Hispanic white LLN-PRISm subjects

	Normalized Mutual Information		Silhouette Width	
k =	mean	SD	mean	SD
2	0.8164	0.1912	0.1666	0.0083
3	0.8728	0.1367	0.1633	0.0093
4	0.8792	0.0300	0.1357	0.0052
5	0.6311	0.0975	0.1234	0.0062
6	0.6238	0.1502	0.1090	0.0137
7	0.6609	0.0670	0.1250	0.0099

(b) Results for African American LLN-PRISm subjects

	Normalized Mutual Information		Silhouette Width	
k	mean	SD	mean	SD
2	0.6547	0.2810	0.1370	0.0409
3	0.6119	0.1646	0.1183	0.0422
4	0.9169	0.0982	0.1435	0.0349
5	0. 8312	0.1433	0.1335	0.0372
6	0.7622	0.0664	0.1067	0.0330
7	0.6502	0.0589	0.0934	0.0223

Additional File – Table S9. Results of unsupervised k-means clustering of (a) non-Hispanic white and (b) African American subjects with *P*reserved *R*atio *I*mpaired *S*pirometry by lower limits of normal criteria.

	(a) Non-Hispanic White subjects				(b) African American subjects			
Feature	Cluster 1	Cluster 2	Cluster 3	Cluster 4	Cluster 1	Cluster 2	Cluster 3	Cluster 4
n	163	171	157	190	75	61	72	89
Age	56.8 (8.5)	65.0 (7.5)	61.7 (8.8)	58.2 (7.6)	52.5 (4.8)	56.4 (6.6)	51.4 (4.4)	53.3 (5.0)
Sex (% male)	47.2	67.8	61.2	44.7	60.0	70.5	34.7	59.6
Body Mass Index	33.6 (6.9)	30.3 (6.3)	35.5 (6.8)	28.6 (5.3)	30.6 (5.9)	27.7 (5.2)	42.3 (6.0)	29.4 (5.5)
Current smoker	52.2	32.8	41.4	70.5	85.3	82.0	76.4	87.6
Pack-years	44.0 (25.3)	53.7 (35.0)	52.9 (29.6)	47.1 (20.1)	34.3 (19.9)	42.5 (22.1)	35.8 (18.0)	39.7 (20.1)
FEV_1 % predicted	71.9 (5.1)	68.7 (6.6)	59.0 (8.1)	71.8 (4.7)	69.2 (5.2)	66.1 (6.8)	66.3 (5.8)	60.7 (8.7)
FEV_1/FVC	0.80 (0.04)	0.70 (0.04)	0.72 (0.04)	0.73 (0.03)	0.83 (0.04)	0.73 (0.04)	0.77 (0.04)	0.74 (0.04)
$FEF_{25\text{-}75}$	2.30 (0.65)	1.35 (0.50)	1.21 (0.38)	1.59 (0.47)	2.44 (0.75)	1.54 (0.52)	1.56 (0.49)	1.37 (0.40)
Resting O2 saturation	96.2 (2.3)	96.1 (2.0)	95.2(2.8)	96.5 (2.0)	97.4 (1.6)	96.8 (2.6)	96.4 (2.2)	96.8 (2.9)
TLC_{CT}* % predicted	76.5 (9.1)	88.8 (11.1)	74.8 (11.5)	91.9 (9.7)	67.0 (10.3)	80.4 (11.0)	81.6 (11.7)	67.3 (11.1)
Percent emphysema	0.9 (1.0)	5.2 (3.8)	1.1 (1.3)	0.7 (0.5)	0.4 (0.4)	4.2 (5.5)	1.2 (1.2)	0.5 (0.5)
Segmental Wall Area %	62.0 (2.4)	60.7 (2.4)	64.7 (2.6)	61.7 (2.5)	61.7 (2.5)	60.5 (3.1)	64.6 (2.4)	65.5 (2.6)
Percent Gas Trapping	7.1 (6.0)	21.4 (10.1)	12.1 (8.9)	9.9 (8.3)	6.7 (7.7)	19.4 (13.8)	11.3 (8.4)	7.9 (6.4)
Diabetes Mellitus	26.4	18.7	38.9	13.2	24.0	23.0	37.5	18.0
Hypertension	41.7	52.6	59.2	36.8	46.7	54.1	61.1	48.3
Hyperlipidemia	47.2	57.9	58.6	50.5	37.3	32.8	34.7	23.6
Chronic bronchitis	20.3	15.2	29.3	22.6	9.3	18.0	15.3	16.9
Bronchodilator Response†	11.7	13.5	18.7	14.9	12.2	6.8	15.3	20.9
MMRC‡	1.20 (1.38)	1.18 (1.32)	1.99 (1.41)	1.17 (1.35)	1.44 (1.56)	1.49 (1.44)	2.39 (1.49)	1.56 (1.54)
6 minute walk distance	1380.8 (358.6)	1381.2 (336.4)	1116.9 (390.3)	1395.5 (310.2)	1298.8 (338.4)	1282.7 (291.3)	1030.5 (353.4)	1206.1 (339.4)

<u>Additional File – Table S10</u> – Candidate gene testing in fixed-threshold Preserved Ratio Impaired Spirometry (PRISm) subgroups.

SNP	Closest Gene	Disease Association in Literature	ANOVA p-value in NHW	ANOVA p-value in AA
rs10811661	*CDKN2B*	Diabetes	NS	NS
rs2237892	*KCNQ1*	Diabetes	NS	NS
rs2283228	*KCNQ1*	Diabetes	NS	NS
rs4402960	*IGF2BP2*	Obesity, diabetes	NS	NS
rs8050136	*FTO*	Obesity, diabetes	NS	0.05
rs9939609	*FTO*	Obesity	NS	NS
rs10146997	*NRXN3*	Central obesity, substance abuse	NS	NS
rs10198628	*TRIB2*	Pericardial fat volume	NS	NS
rs161976	*BICD1*	COPD/emphysema	NS	NS
rs10844154	*BICD1*	COPD/emphysema	NS	NS
rs13180	*IREB2*	COPD	NS	NS
rs8034191	*AGPHD; CHRNA3/5*	COPD	NS	NS
rs1051730	*CHRNA3*	COPD	NS	NS
rs12504628	*HHIP*	COPD	NS	NS
rs1980057	*HHIP*	COPD	NS	0.02
rs7671167	*FAM13A*	COPD	NS	NS
rs2609255	*FAM13A*	ILD/IPF	NS	NS
rs2736100	*TERT*	ILD/IPF	NS	NS
rs868903	*MUC5B*	ILD/IPF	NS	NS
rs35705950	*MUC5B*	ILD/IPF	NS	NS
rs2070600	*AGER*	Lung function	NS	NS

NS = “Not significant”

Additional Figures

Additional File - Figure S1. Distribution of spirometry in COPDGene by lower limit of normal criteria.

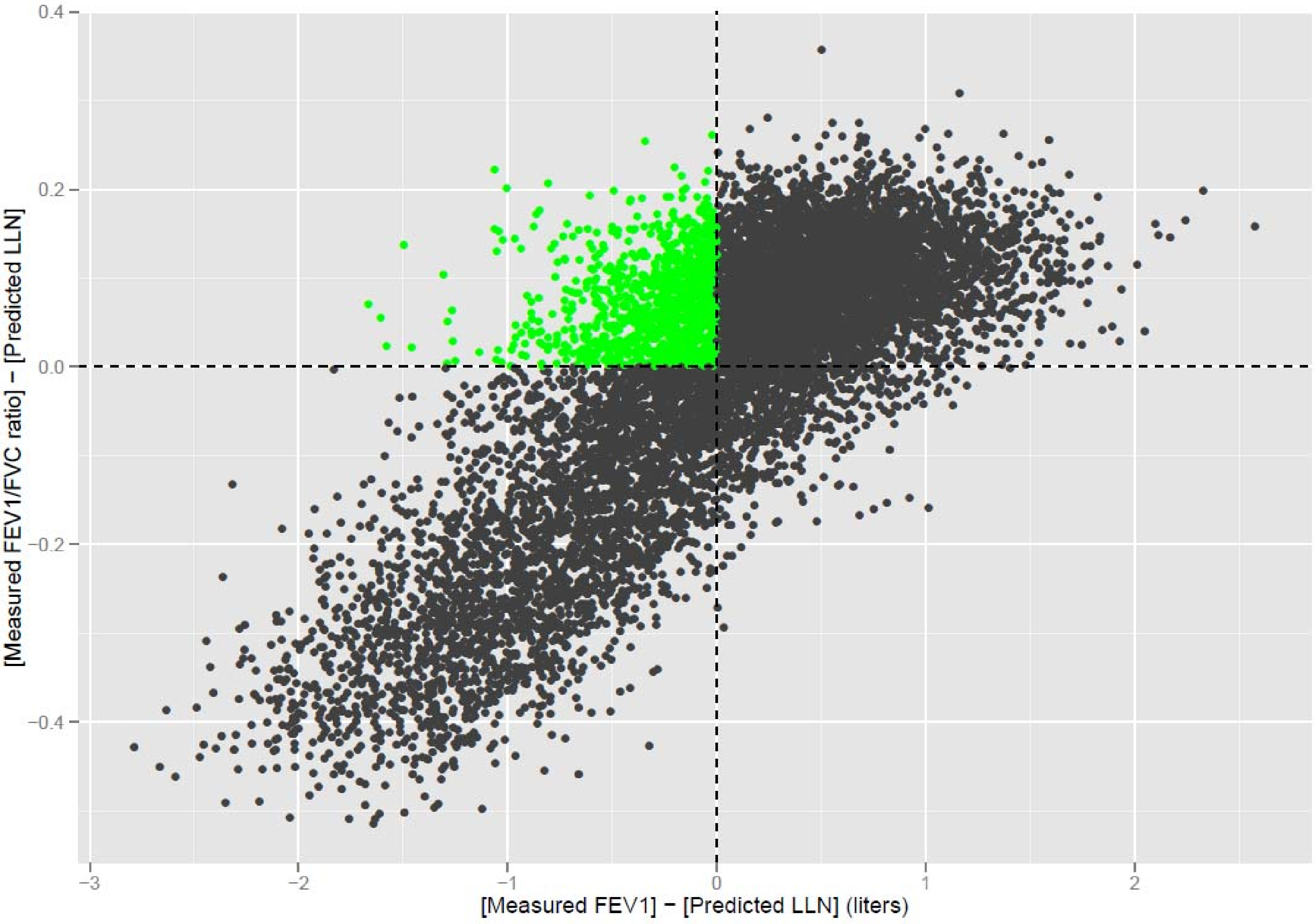

The difference between measured FEV_1 and the LLN-predicted FEV_1 is plotted on the x-axis while the difference between measured FEV_1/FVC ratio and the LLN-predicted ratio is plotted on the y-axis. Dashed lines indicate where the difference between measured values and LLN = 0 (i.e. LLN) and delineate subjects with LLN – PRISm (highlighted in green) in the upper left quadrant, LLN-controls (upper right quadrant), and LLN-COPD (lower left quadrant).

<u>Additional File - Figure S2</u>– Overlap between fixed threshold-defined (red oval, n_{total} = 1,257) and lower limit of normal (LLN)-defined (blue oval, n_{total} – 1,082) Preserved Ratio Impaired Spirometry (PRISm) cohorts in COPDGene.

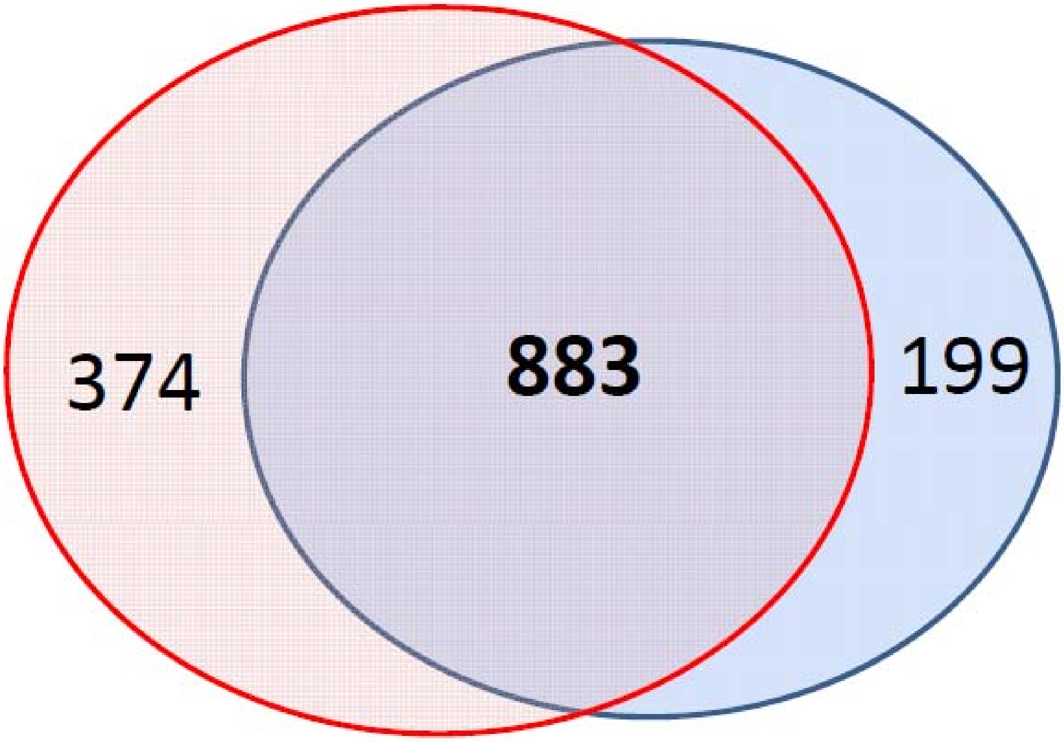

<u>Additional File </u> - Figure S3. Reclassification of PRISm subjects by application of (a) lower limit of normal (LLN) or (b) fixed threshold criteria.

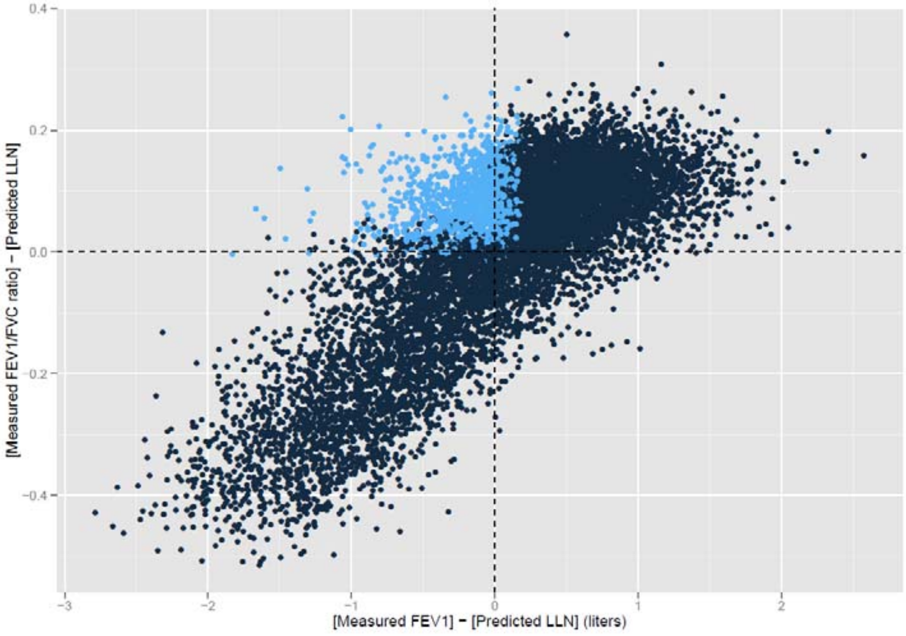

(a)

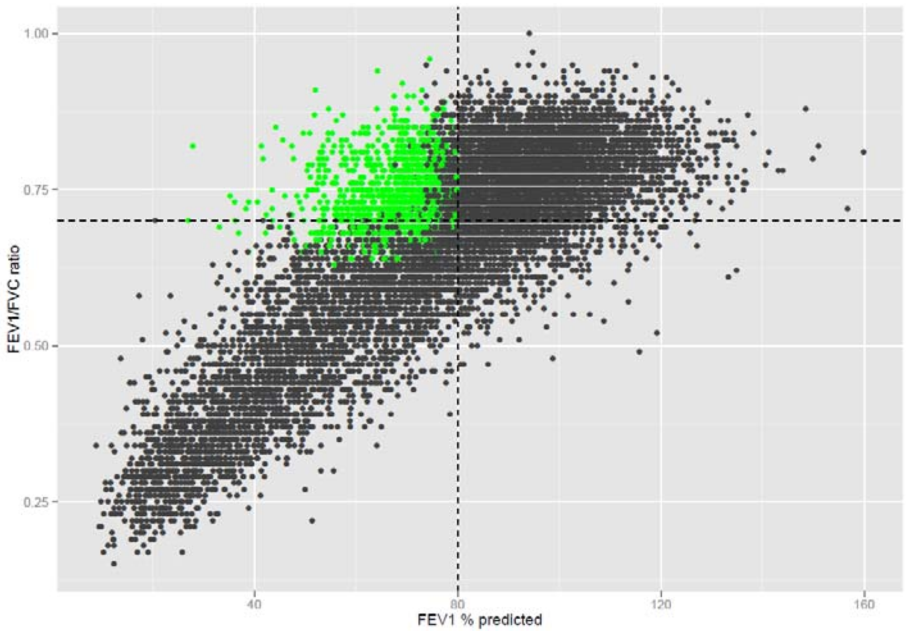

(b)

For both panels, dashed lines represent the thresholds used to define PRISm (upper left quadrant), COPD (lower left quadrant), and control subjects (upper right quadrant). In panel (a) subjects with *fixed threshold defined* PRISm are plotted (in blue) against LLN-delineated thresholds. A large number of fixed threshold PRISm subjects are thus seen to be classified as control subjects by LLN thresholds. In panel (b) *LLN-defined* PRISm subjects (in green) are plotted against fixed-threshold criteria – a large number of LLN-PRISm subjects are classified as COPD subjects using fixed-threshold criteria.

Additional File - Figure S4 – Plot of mean single nucleotide polymorphism (SNP) intensities located on the X (x-axis) and Y (y-axis) chromosomes. Each point represents one subject; color of point represents self-reported gender (blue = male, pink = female). Six Klinefelter syndrome (47XXY) subjects were putatively identified (red oval).

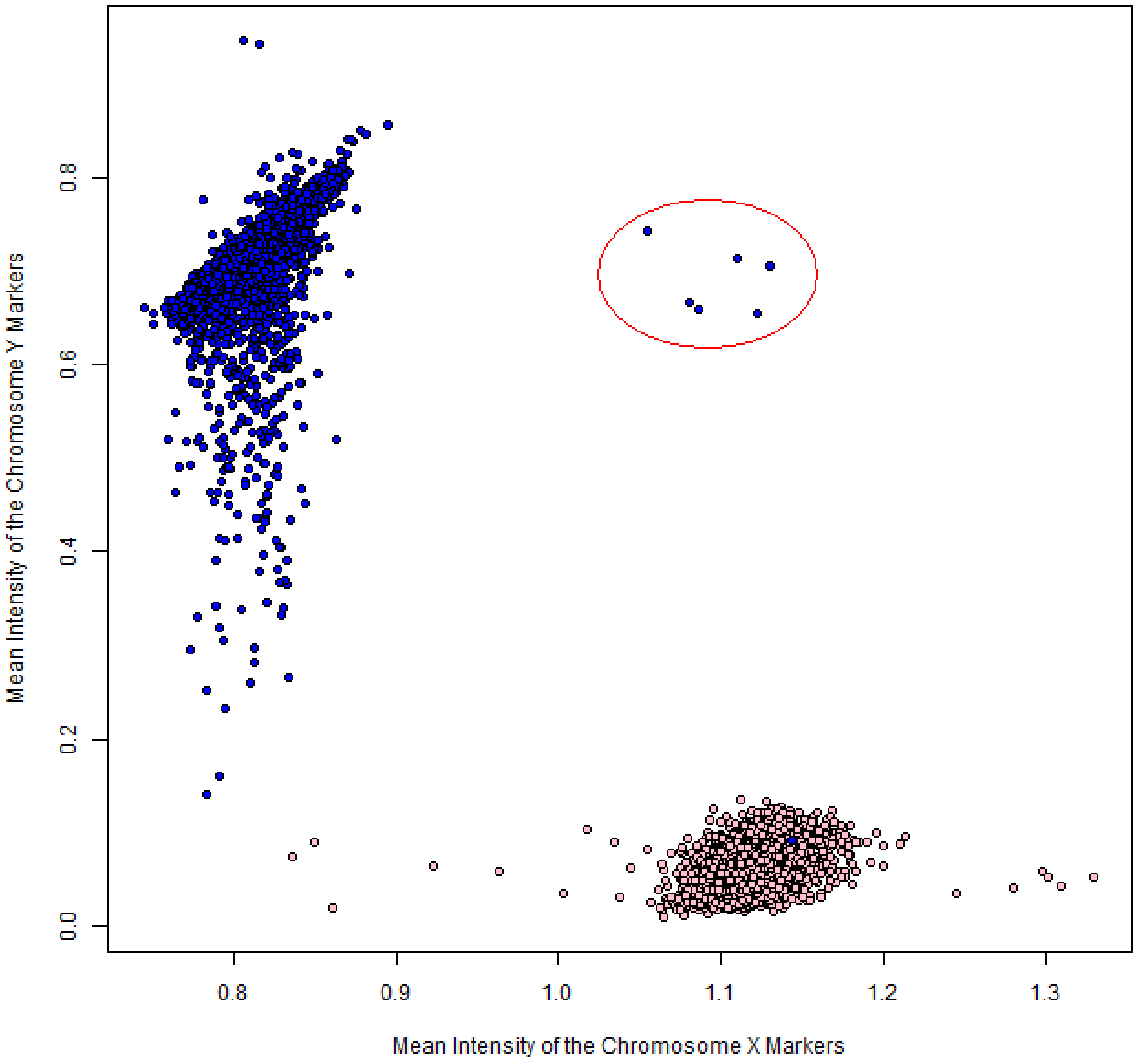

<u>Additional File - Figure S5</u> - (a) Quantile-quantile plot of expected (x-axis) and actual (y-axis) p-values and (b) Manhattan plot of actual p-values (y-axis) by chromosomal location (x-axis) for meta-analysis of fixed threshold-defined Preserved Ratio Impaired Spirometry (PRISm) subjects versus control subjects genome-wide association study.

(a)

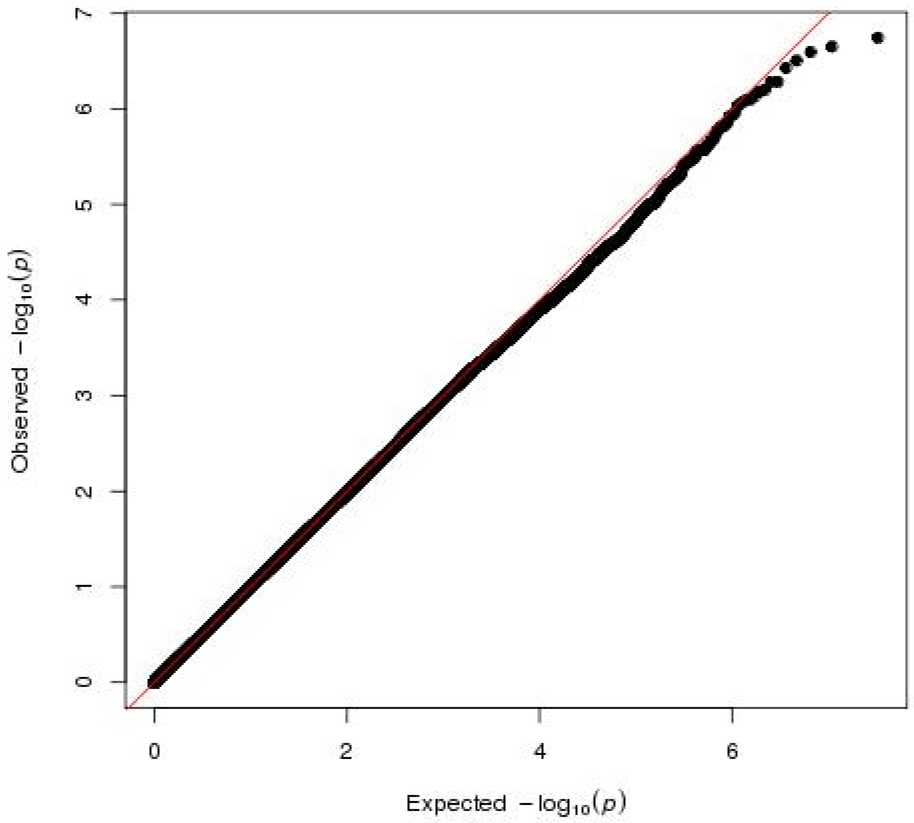

(b)

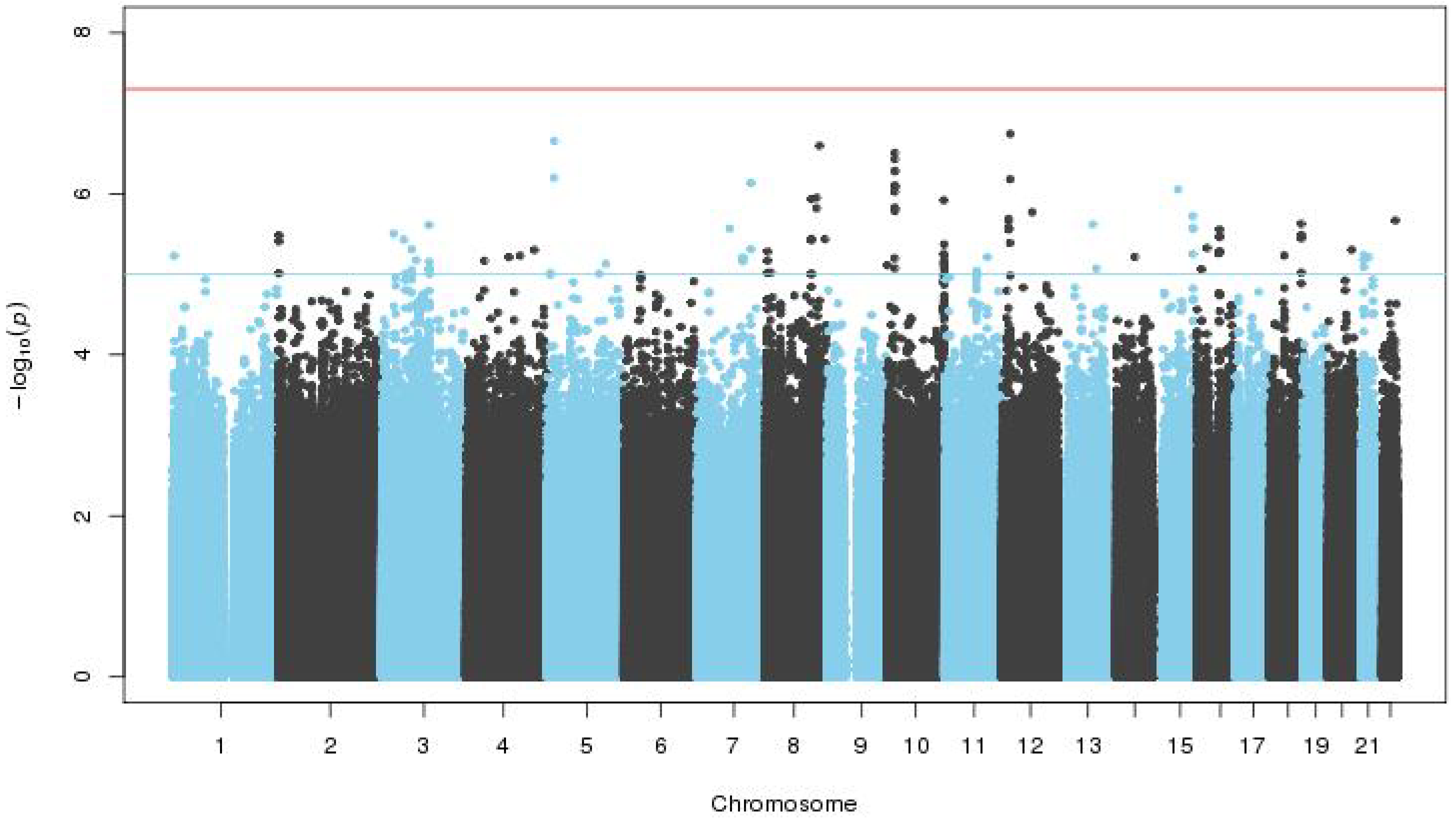

<u>Additional File </u> - Figure S6. Clustering results of NHW (solid lines) and AA (hatched lines) subjects with fixed-threshold defined *P*reserved *R*atio *I*mpaired *S*pirometry.

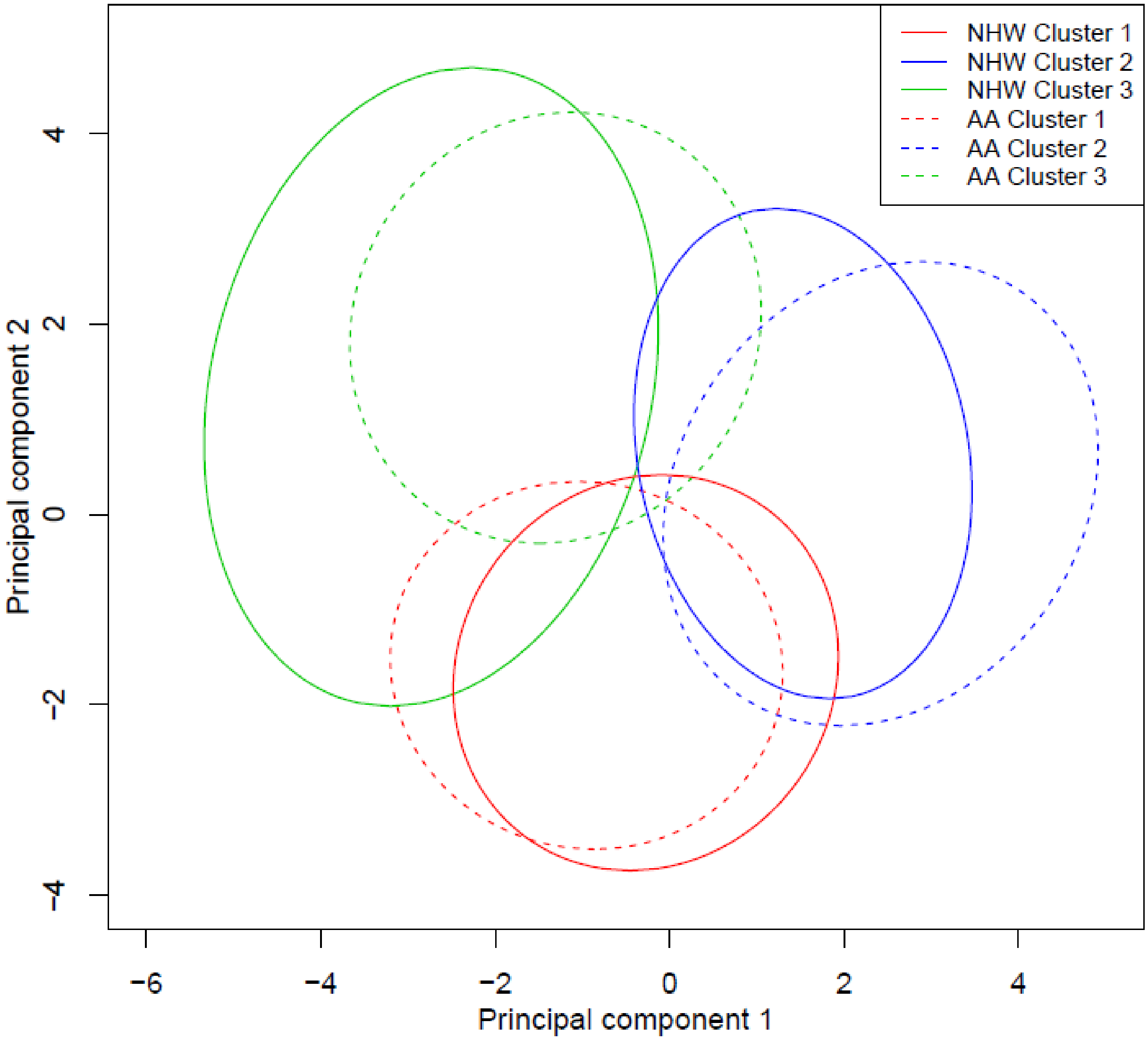

Additional File - **Figure S7.** Results of unsupervised k-means clustering in (a) non-Hispanic white and (b) African American subjects with lower limit of normal defined *P*reserved *R*atio *I*mpaired *S*pirometry.

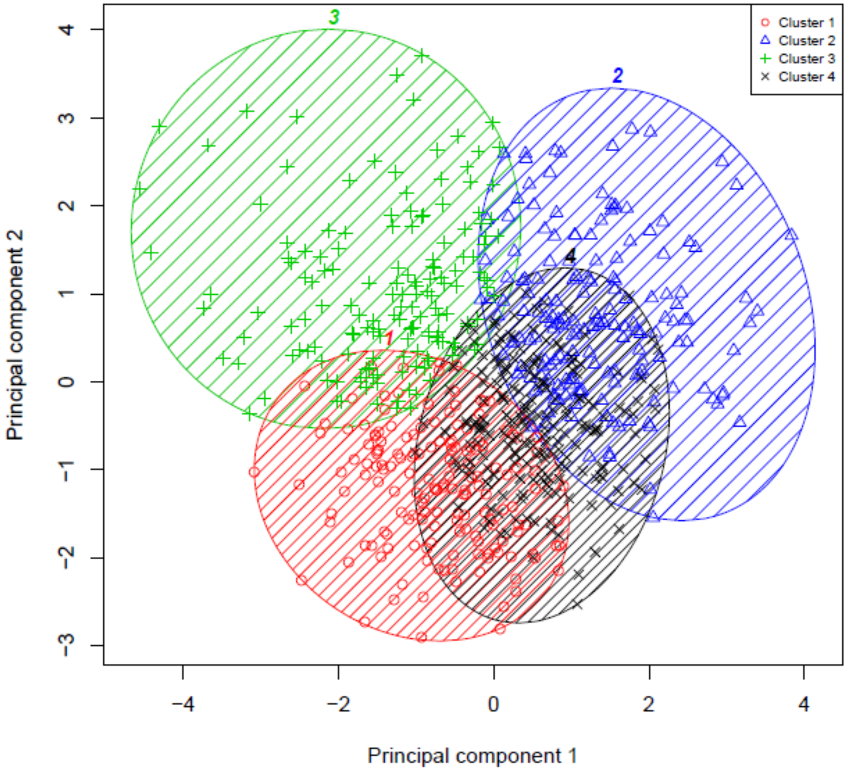

(a)

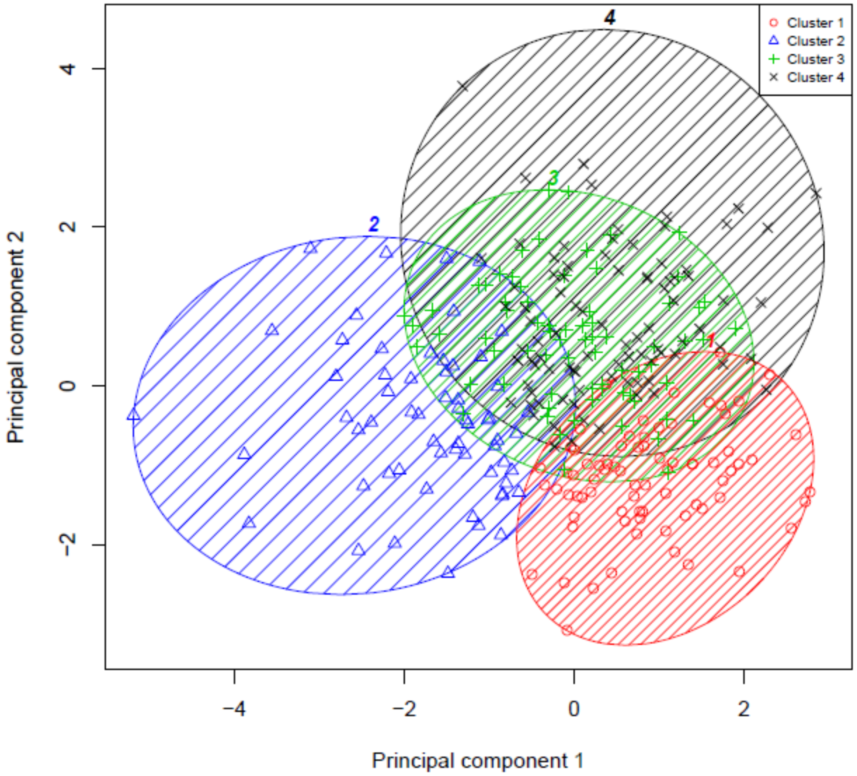

(b)

Additional File – Figure S8. Clustering results of NHW (solid lines) and AA (hatched lines) subjects with lower limit of normal- defined *P*reserved *R*atio *I*mpaired *S*pirometry.

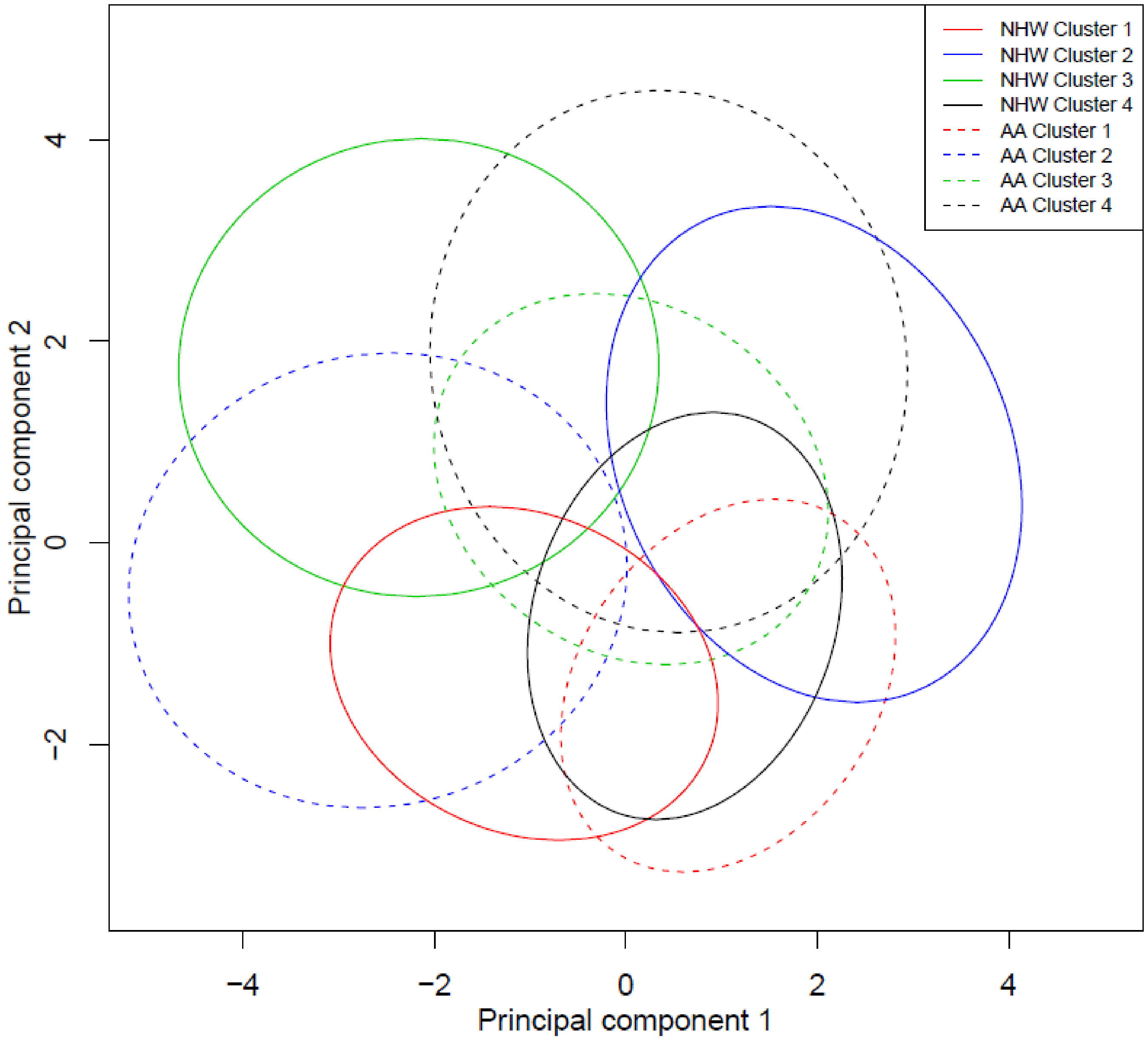

<u>Additional File - Figure S9</u> – Minor (risk) allele frequency of rs8050136 (*FTO*) by race and cluster in fixed threshold-defined *P*reserved *R*atio *I*mpaired *S*piro*m*etry (PRISm) subjects.

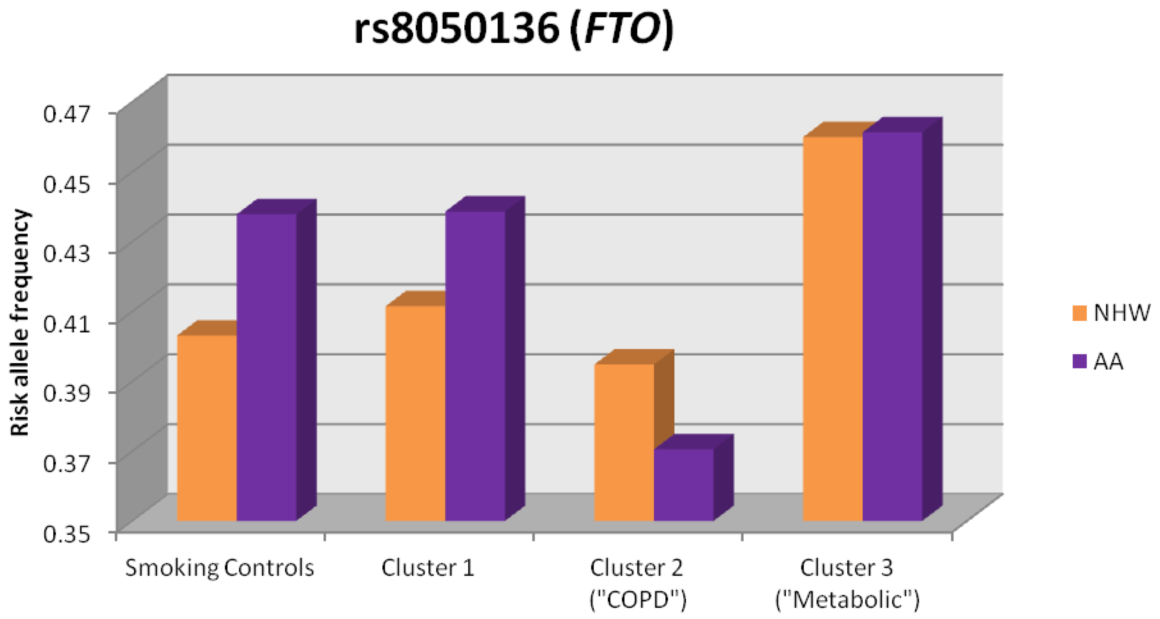

NHW = non-Hispanic white, AA = African American

Additional File - Figure S10– Prevalence of overweight and obesity in the United States[8] from 1960-2010 relative to the prevalence of *P*reserved *R*atio *I*mpaired *S*piro*m*etry (PRISm)[9-19].

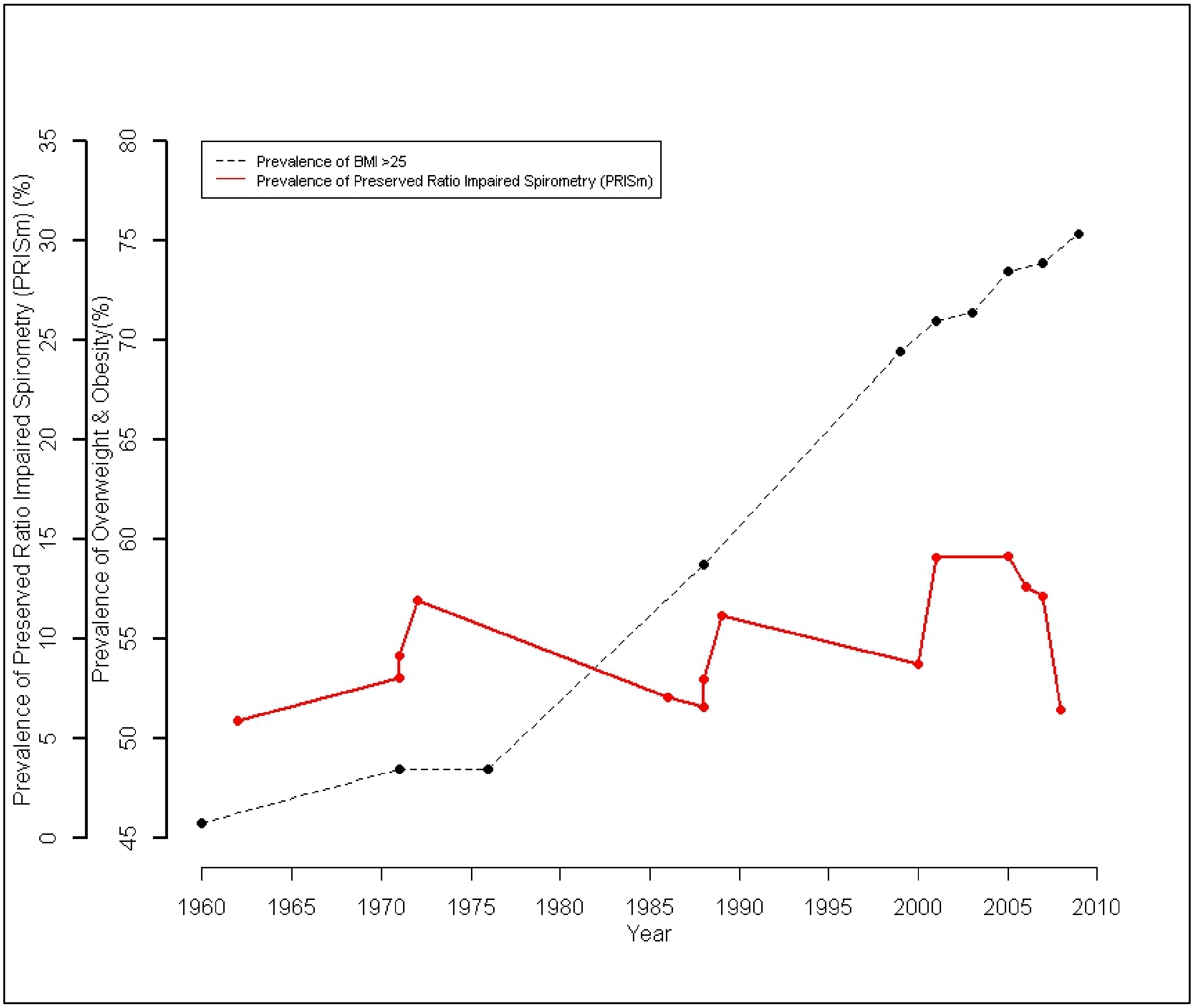

Additional Files – References

1. Pellegrino R, Viegi G, Brusasco V, Crapo RO, Burgos F, Casaburi R, Coates A, van der Grinten CP, Gustafsson P, Hankinson J *et al*: **Interpretative strategies for lung function tests**. *Eur Respir J* 2005, **26**(5):948-968.
2. Stocks J, Quanjer PH: **Reference values for residual volume, functional residual capacity and total lung capacity. ATS Workshop on Lung Volume Measurements. Official Statement of The European Respiratory Society**. *Eur Respir J* 1995, **8**(3):492-506.
3. Grydeland TB, Dirksen A, Coxson HO, Pillai SG, Sharma S, Eide GE, Gulsvik A, Bakke PS: **Quantitative computed tomography: emphysema and airway wall thickness by sex, age and smoking**. *Eur Respir J* 2009, **34**(4):858-865.
4. **SAS/STAT(R) 9.2 User's Guide, Second Edition** [http://support.sas.com/documentation/cdl/en/statug/63033/HTML/default/viewer.htm#statug_logistic_sect052.htm]
5. **COPDGene GWAS Quality Control and Methodology** [http://www.copdgene.org/sites/default/file/GWAS_QC_Methodology_20121115.pdf]
6. **R Core Team. R: A language and environment for statistical computing.** [http://www.R-project.org/.2012]
7. Hartigan JA, Wong MA: **A K-means clustering algorithm**. *Applied Statistics* 1979, **28**:100-108.
8. **Prevalence of Overwieght, Obesity, and Extreme Obesity Among Adults: United States, Trends 1960-1962 Through 2009-2010.** [http://www.cdc.gov/nchs/data/hestat/obesity_adult_09_10/obesity_adult_09_10.htm]
9. Ford ES, Mannino DM, Wheaton AG, Giles WH, Presley-Cantrell L, Croft JB: **Trends in the prevalence of obstructive and restrictive lung function among adults in the United States: findings from the National Health and Nutrition Examination surveys from 1988-1994 to 2007-2010**. *Chest* 2013, **143**(5):1395-1406.
10. Ford ES, Mannino DM, Zhao G, Li C, Croft JB: **Changes in mortality among US adults with COPD in two national cohorts recruited from 1971-1975 and 1988-1994**. *Chest* 2012, **141**(1):101-110.
11. Guerra S, Sherrill DL, Venker C, Ceccato CM, Halonen M, Martinez FD: **Morbidity and mortality associated with the restrictive spirometric pattern: a longitudinal study**. *Thorax* 2010, **65**(6):499-504.
12. Hickson DA, Liu J, Bidulescu A, Burchfiel CM, Taylor HA, Petrini MF: **Pericardial fat is associated with impaired lung function and a restrictive lung pattern in adults: the Jackson Heart Study**. *Chest* 2011, **140**(6):1567-1573.
13. Kohler D, Fischer J, Raschke F, Schonhofer B: **Usefulness of GOLD classification of COPD severity**. *Thorax* 2003, **58**(9):825.
14. Mannino DM, Doherty DE, Sonia Buist A: **Global Initiative on Obstructive Lung Disease (GOLD) classification of lung disease and mortality: findings from the Atherosclerosis Risk in Communities (ARIC) study**. *Respir Med* 2006, **100**(1):115-122.
15. Mannino DM, Ford ES, Redd SC: **Obstructive and restrictive lung disease and functional limitation: data from the Third National Health and Nutrition Examination**. *J Intern Med* 2003, **254**(6):540-547.
16. Mannino DM, McBurnie MA, Tan W, Kocabas A, Anto J, Vollmer WM, Buist AS: **Restricted spirometry in the Burden of Lung Disease Study**. *Int J Tuberc Lung Dis* 2012, **16**(10):1405-1411.
17. Soriano JB, Miravitlles M, Garcia-Rio F, Munoz L, Sanchez G, Sobradillo V, Duran E, Guerrero D, Ancochea J: **Spirometrically-defined restrictive ventilatory defect: population variability and individual determinants**. *Prim Care Respir J* 2012, **21**(2):187-193.
18. Vaz Fragoso CA, Gill TM, McAvay G, Yaggi HK, Van Ness PH, Concato J: **Respiratory impairment and mortality in older persons: a novel spirometric approach**. *J Investig Med* 2011, **59**(7):1089-1095.
19. Wan ES, Hokanson JE, Murphy JR, Regan EA, Make BJ, Lynch DA, Crapo JD, Silverman EK: **Clinical and radiographic predictors of GOLD-unclassified smokers in the COPDGene study**. *Am J Respir Crit Care Med* 2011, **184**(1):57-63.
